# HGF/c-Met/β1-integrin signalling axis induces tunneling nanotubes in A549 lung adenocarcinoma cells

**DOI:** 10.26508/lsa.202301953

**Published:** 2023-08-07

**Authors:** Griselda Awanis, Salonee Banerjee, Robert Johnson, Sathuwarman Raveenthiraraj, Aya Elmeligy, Derek Warren, Jelena Gavrilovic, Anastasia Sobolewski

**Affiliations:** 1 School of Pharmacy, University of East Anglia , Norwich, UK; 2 School of Biological Sciences, University of East Anglia , Norwich, UK

## Abstract

Tunneling nanotubes (TNTs) are thin cytoplasmic extensions involved in long-distance intercellular communication and can transport intracellular organelles and signalling molecules. In cancer cells, TNT formation contributes to cell survival, chemoresistance, and malignancy. However, the molecular mechanisms underlying TNT formation are not well defined, especially in different cancers. TNTs are present in non-small cell lung cancer (NSCLC) patients with adenocarcinoma. In NSCLC, hepatocyte growth factor (HGF) and its receptor, c-Met, are mutationally upregulated, causing increased cancer cell growth, survival, and invasion. This study identifies c-Met, β1-integrin, and paxillin as novel components of TNTs in A549 lung adenocarcinoma cells, with paxillin localised at the protrusion site of TNTs. The HGF-induced TNTs in our study demonstrate the ability to transport lipid vesicles and mitochondria. HGF-induced TNT formation is mediated by c-Met and β1-integrin in conjunction with paxillin, followed by downstream activation of MAPK and PI3K pathways and the Arp2/3 complex. These findings demonstrate a potential novel approach to inhibit TNT formation through targeting HGF/c-Met receptor and β1-integrin signalling interactions, which has implications for multi-drug targeting in NSCLC.

## Abstract (precis)

Interactions between hepatocyte growth factor (HGF), the c-Met receptor, and β1-integrin induce tunneling nanotubes in A549 cells; paxillin and the downstream MAPK and PI3K pathways and the Arp2/3 complex drive HGF-induced TNT formation.

## Introduction

Non-small cell lung cancer (NSCLC) accounts for 85% of all lung cancer cases, with adenocarcinoma being the most common form of NSCLC. It is associated with high mortality rates as the majority of patients are diagnosed with advanced metastatic cancer and demonstrate a lack of response to chemotherapy (Molina et al, 2008). Therefore, it is important to understand how lung adenocarcinoma cells communicate with each other within the tumour microenvironment (TME) to promote metastasis and chemoresistance. One mode of cell-to-cell communication is through tunneling nanotubes (TNTs). TNTs are thin cellular extensions that can extend over 100 μm in length and connect cells together over long distances (Rustom et al, 2004; Ariazi et al, 2017). TNTs are non-adherent, open-ended, and F-actin-based cytoplasmic protrusions with microtubules expressed in thicker TNTs (Onfelt et al, 2006). TNTs can transport mitochondria, Golgi vesicles, miRNA, and signalling molecules between cells (Wang et al, 2011; Lou et al, 2012; Thayanithy et al, 2014; Wang & Gerdes, 2015; Lu et al, 2017, 2019) and promote cancer progression and resistance to chemotherapeutic agents (Lou et al, 2012; Pasquier et al, 2013; Desir et al, 2018; Wang et al, 2018). TNTs can occur between different cell types or between cells of the same type and have been observed in lung adenocarcinoma A549 cells (Wang et al, 2012, 2021; Kumar et al, 2017; Dubois et al, 2018). Importantly, TNTs have also been observed in vivo in human lung adenocarcinoma tissue, where mitochondria transport was observed (Lou et al, 2012). Despite the important role of TNTs in cancer, the regulatory mechanisms and signalling pathways associated with TNT formation are poorly defined, especially in lung adenocarcinoma.

Studies have mainly focused on the molecular mechanisms involved in stress-mediated TNT formation, which occur through the Akt/PI3K/mTOR and p38 MAPK signalling pathways (Zhu et al, 2005; Wang et al, 2011). Actin regulators including the Rho GTPases (CDC42 and Rac1) are also implicated in TNT formation (Hase et al, 2009; Gousset et al, 2013; Schiller et al, 2013; Hanna et al, 2017). However, studies on which specific exogenous factors can induce TNTs, especially in lung adenocarcinoma, are lacking, despite the pathophysiological evidence of TNT formation in lung adenocarcinoma cells in vivo (Lou et al, 2012).

The hepatocyte growth factor (HGF)-c-Met receptor signalling axis is known to be dysregulated in NSCLC (Ichimura et al, 1996; Olivero et al, 1996; Tretiakova et al, 2011). Moreover, HGF is a pleiotropic cytokine which regulates morphogenesis, growth, epithelial to mesenchymal transformation, and migration (Stoker et al, 1987; Yanagita et al, 1993; Singh-Kaw et al, 1995; Ohmichi et al, 1996). Therefore, HGF may act as a potential exogenous factor for inducing actin-rich TNTs in lung adenocarcinoma. The HGF receptor, c-Met, is a receptor tyrosine kinase and proto-oncogene (Sonnenberg et al, 1993), which induces multiple signalling cascades including the MAPK, PI3K, and FAK pathways that are dysregulated after mutation to the c-Met oncogene (Zhang & Vande Woude, 2003; Corso et al, 2005; Gherardi et al, 2006). Furthermore, in addition to paracrine signalling of the c-Met receptor on NSCLC cells through HGF secretion by stromal fibroblasts, lung adenocarcinoma cells can also undergo autocrine signalling, raising the possibility that HGF could also play a role in TNT formation between cancer cells (Masuya et al, 2004; Nakamura et al, 2007). Other receptors can crosstalk with the HGF/c-Met receptor-signalling axis in lung cancer, in particular β1-integrin (Ju & Zhou, 2013; Barrow-McGee et al, 2016). HGF activation induces integrin clustering, the recruitment of FAK and paxillin, and the transduction of downstream MAPK, PI3K, and Rho GTPase pathways which are important in cancer cell growth, invasion, and metastasis (Lai et al, 2000; Liu et al, 2002; Ishibe et al, 2003, 2004). However, it is not known whether exogenous factors such as HGF can induce TNTs in lung adenocarcinoma cells.

Therefore, the aim of this study was to determine whether HGF/c-Met/β1-integrin signalling axis regulates TNT formation in A549 lung adenocarcinoma cells. This work demonstrates a novel role for the co-activation of HGF/c-Met and β1-integrin in regulating TNT formation in A549 cells via paxillin and downstream Arp2/3 complex, MAPK, and PI3K pathways. C-Met, β1-integrin, and paxillin were identified as novel components of TNTs in A549 lung adenocarcinoma cells.

## Results

### HGF induces the formation of TNT-like structures in A549 lung adenocarcinoma cells

**Figure 1.**
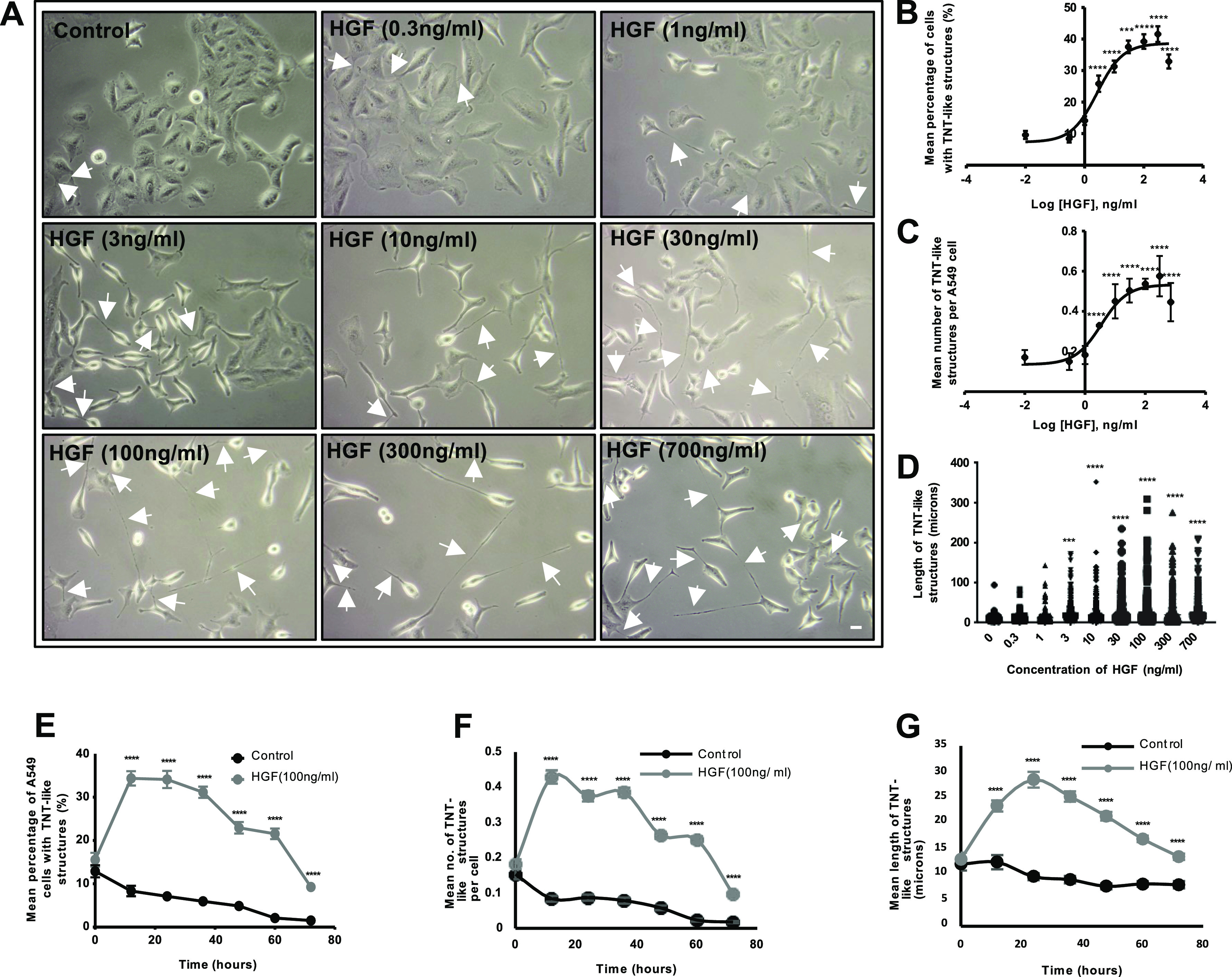
HGF induces TNT-like structures in A549 lung adenocarcinoma cells in a time- and concentration-dependent manner. **(A)** Representative phase contrast images demonstrate an increase in TNT-like structures (white arrows) with increasing concentrations of HGF (0–700 ng/ml). Phase contrast images were captured using 10x objective lens on an inverted microscope. Scale bar: 10 μm. **(B, C, D)** The phase contrast images were quantified for TNT-like structures, and the HGF log concentration-response curve displays the mean percentage (B), number of TNT-like structures per cell (C), and length of TNT-like structures (D). **(E, F, G)** The line graphs show the dose-dependent increase in mean percentage (E), number (F), and length (G) of the HGF (100 ng/ml)-induced TNT-like structures over a 72-h time period when compared with control. **(E, G)** The maximal effect was observed at the 24th h time point for mean percentage (E) and mean length (G). Values are expressed as mean ± SEM, n = 3, with at least 600 cells analysed per condition. ***P < 0.001 and ****P < 0.0001 when compared with control.

**Figure S1.**
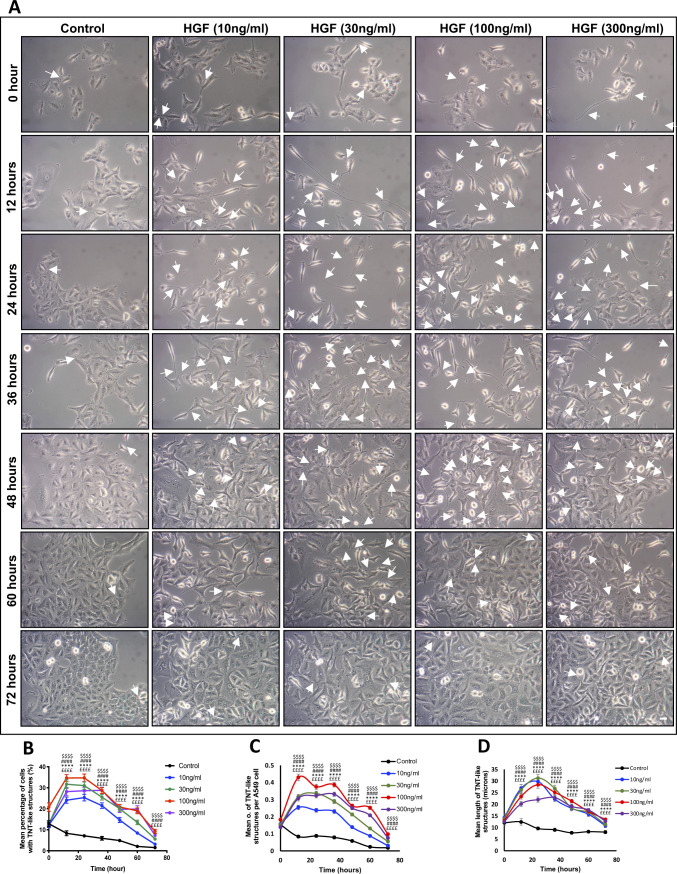
HGF induces TNT-like structures in A549 lung adenocarcinoma cells in a time-dependent manner. **(A)** Representative white light images demonstrate an increase in TNT-like structures (white arrows) with increasing concentrations of HGF (0–300 ng/ml) over 72 h. White light images were captured using 10x objective lens on an inverted microscope. Scale bar: 10 μm. **(B, C, D)** The white light images were quantified for TNT-like structures, and the line graphs showed a dose-dependent increase in the mean (B), number (C), and length (D) of the HGF-induced TNT-like structures between control and the different concentrations of HGF-treated cells over a 72-h time period. The maximal effect was observed at the 24th h time point for the mean percentage and length of the HGF-induced TNT-like structures. Values are expressed as mean ± SEM, n = 3, with at least 1,500 cells analysed per condition. ££££*P* < 0.0001 when comparing control to 10 ng/ml, ****P < 0.0001 when comparing control to 30 ng/ml, ####*P* < 0.0001 when comparing control to 100 ng/ml, $$$$*P* < 0.0001 when comparing control to 300 ng/ml across all the time points, by one-way ANOVA and Tukey’s multiple comparison test.

To determine the effect of HGF on TNT formation of A549 cells, phase contrast images were captured on an inverted microscope after 24-h treatment of HGF (0–700 ng/ml) (Fig 1A). HGF induced thin cellular protrusions, narrow at the base and spanning various lengths to connect to distant cells. Therefore, we termed these protrusions TNT-like structures, as they display morphology akin to TNTs. Between 3 and 30 ng/ml, HGF induced A549 cells to scatter and display characteristic elongated morphology. TNT-like structures also began to form as the A549 cells exhibited filopodia-like extensions. At higher concentrations between 100 and 700 ng/ml, TNT-like extensions begin to lengthen across the field of view. To quantify the formation of TNT-like structures, the mean percentage of cells with TNT-like structures, mean number of TNT-like structures per cell, and the length of TNT structures were measured. The log concentration-response curve shows HGF induces a dose-dependent significant (n = 3, ****P < 0.0001) increase in mean percentage, mean number per cell, and length of TNT-like structures, with a maximal concentration reached at 100 ng/ml plateauing through 300 and 700 ng/ml (Fig 1B–D, respectively). However, TNT-like structures were observed at lengths spanning several hundred microns, reaching up to 350 μm in our study (Fig 1D). To identify the optimal time point for TNT observation, a 72-h time course experiment was conducted for HGF at its maximal concentration (100 ng/ml) compared with the control. The concentration for this time course experiment was determined through additional studies (Fig S1A–D). The line graph (Fig 1E–G) displayed a dome-shaped response curve in mean percentage, number per cell, and length of TNT-like structures over 72 h. The maximal time point was reached at the 24th h for two of the parameters: mean percentage (Fig 1E) and mean length (Fig 1G) of TNT-like structures. Therefore, the maximal concentration (100 ng/ml) and time point (24 h) induced by HGF were used for subsequent experiments.

We next determined whether the TNT-like structures identified in our phase contrast images also formed connections with other cells via time-lapse microscopy analysis. In control cells, 70.9% ± 24.1 of TNT-like structures quantified at the 24-h time point formed a connected TNT in the preceding 24 h. In HGF-treated cells, 73.9% ± 7.34 of TNT-like structures at 24 h formed a connected TNT in the preceding 24 h (HGF N = 313; control N = 40, n = 3 experiments).

### HGF-induced TNTs express F-actin and α-tubulin

**Figure 2.**
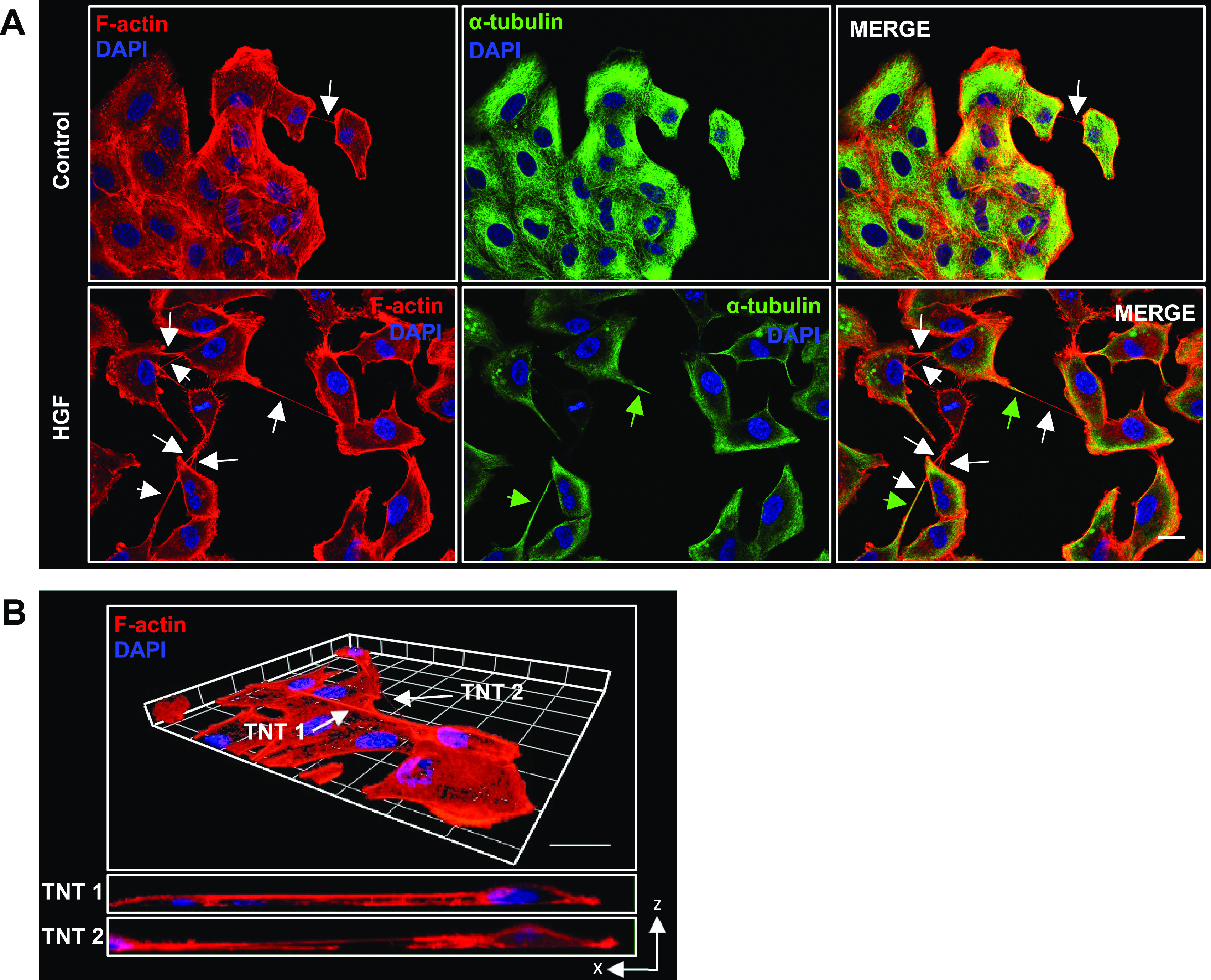
Characterisation of TNT-like structures using immunofluorescent labelling and confocal microscopy. **(A)** Representative confocal images for control and 24 h HGF-treated cells (100 ng/ml) labelled with (A) F-actin (red) and α-tubulin (green). White arrows denote TNT structures, and the dotted area denotes a zoomed area. **(B)** Three-dimensional reconstruction of F-actin–positive non-adherent TNT. The three-dimensional reconstruction was acquired using the ZEN lite software from 49 sections of z-stacked images (with Z step of 0.27 μm). The maximum xz projection images demonstrate two TNTs hovering above the surface of the substratum. Scale bar, 20 μm.

TNTs structurally comprise F-actin with microtubules in the thicker regions of TNTs (Rustom et al, 2004; Onfelt et al, 2006). Immunofluorescent labelling with phalloidin and α-tubulin and subsequent confocal imaging confirmed the TNT-like structures contain F-actin throughout with α-tubulin expressed at the thicker regions (Fig 2A). In addition, TNTs do not adhere to the substratum of the tissue culture surface. We confirmed this important morphological characteristic through confocal z-stack acquisition. The 3D reconstruction and orthogonal view showed HGF-induced TNTs passing over other cells and visible only in higher focal planes, demonstrating their non-adherence (Fig 2B). In summary, the TNT-like structures we observed were non-adherent, expressed F-actin and α-tubulin markers indicating the presence of TNTs.

### HGF-induced TNTs transport mitochondria and lipid vesicles

**Figure 3.**
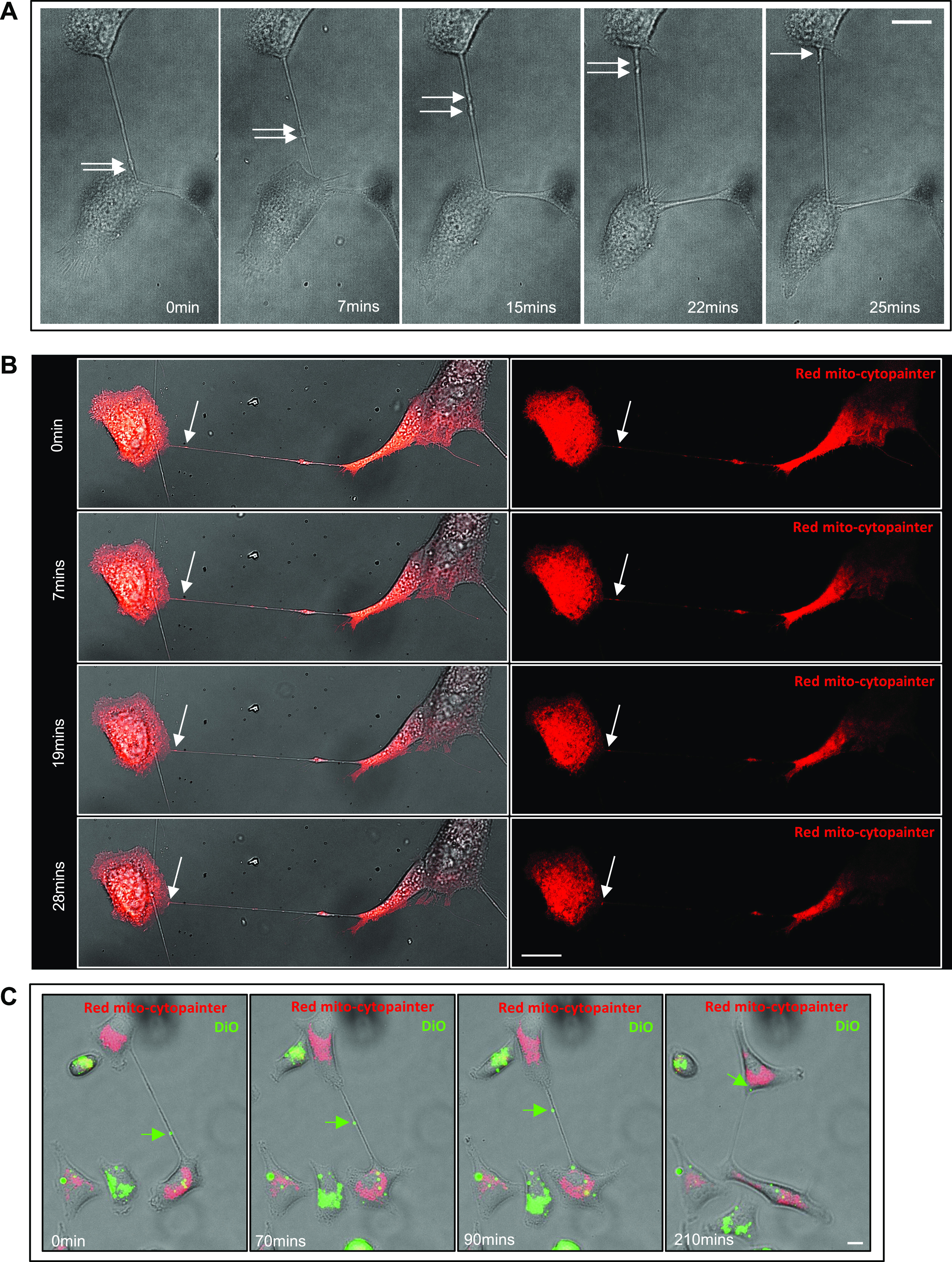
TNTs in A549 cells functionally transmit mitochondria and vesicles. **(A)** Representative confocal DIC images with the indicated time points from a time-lapse movie showing two vesicles (indicated by the white arrows) travelling along a TNT to the cell above. **(B)** Representative confocal DIC-merged fluorescent images with the indicated time points from a time-lapse movie showing mitochondria transfer along a TNT. A549 cells were treated with HGF (100 ng/ml) for 24 h before being loaded with red mitochondrial cytopainter and imaged for live confocal microscopy. Unidirectional mitochondria transfer along the TNT was observed (indicated by the white arrow). **(C)** Representative merged phase contrast and fluorescent image of a DiO-labelled vesicle (indicated by the green arrow) travelling along a TNT towards a red mitochondria-stained cell. Separate populations of A549 cells were loaded with either DiO or red mitochondrial cytopainter and seeded together before HGF treatment and undertaking confocal imaging as above. Bidirectional transfer is suggested through the presence of mitochondria (red) in the DiO (green)-labelled cell below. Scale bar, 20 μm.

TNTs are known to transport organelles, such as mitochondria and vesicles (Rustom et al, 2004; Lou et al, 2012). Live-cell confocal, phase, and epifluorescent microscopy were used to investigate whether HGF-induced TNTs were able to transport mitochondria or vesicles between TNT-connected cells. The phase contrast time-lapse images show two vesicles (white arrows) transferred along a TNT, from a donor cell into the acceptor cell (Fig 3A and Video 1). Further time-lapse analysis showed bidirectional vesicle transfer was observed in 31.9 ± 8.9% of HGF-induced TNTs, while 68.1 ± 8.9% showed unidirectional vesicle transfer (N = 140, n = 3). To visualise mitochondria transfer, A549 cells were treated with HGF and loaded with a red mitochondrial cytopainter before live-cell confocal imaging was undertaken. The sequence of time-lapse images demonstrates mitochondria transfer along TNT-connected cells (Fig 3B, Video 2 and Video 3). Cells were also loaded with either red mitochondrial cytopainter or DiO lipophilic dye (Fig 3C and Video 4). Cells were then seeded together and allowed to adhere before treatment with HGF and live-cell imaging. Fig 3C shows a DiO-labelled vesicle (green arrow) moving along a TNT towards a mitochondria-labelled cell above. Furthermore, the cell below contains both DiO-labelled green vesicles and red mitochondria, suggesting a bidirectional exchange of organelles. HGF-treated A549 cells were also loaded with DiO lipophilic dye alone, and live-cell imaging revealed the unidirectional transfer of two DiO vesicles along a TNT to a recipient cell (Video 5).

Video 1 Time-lapse movie of two vesicles transferred along an HGF-induced TNT. A549 cells were treated with HGF (100 ng/ml) for 24 h before undertaking live-cell imaging. Real-time acquisition of confocal DIC images were captured at 1 min intervals. The time-lapse movie shows unidirectional transfer of two vesicles along a TNT. Scale bar, 20 μm.Download video

Video 2 Time-lapse movie of mitochondria transfer along an HGF-induced TNT. A549 cells were treated with HGF (100 ng/ml) for 24 h and loaded with red mitochondrial cytopainter. Real-time acquisition of merged DIC and fluorescent images was captured at 1 min intervals. The time-lapse movie shows unidirectional mitochondria transfer along the TNT. Scale bar, 20 μm.Download video

Video 3 Time-lapse movie of mitochondria transfer along an HGF-induced TNT. A549 cells were treated with HGF (100 ng/ml) for 24 h and loaded with red mitochondrial cytopainter. Real-time acquisition of fluorescent images alone was captured at 1 min intervals. The time-lapse movie shows unidirectional mitochondria transfer along the TNT. Scale bar, 20 μm.Download video

Video 4 Time-lapse movie of DiO-labelled vesicle transfer along an HGF-induced TNT towards a mitochondria-stained cell. A549 cells were loaded with either DiO or red mitochondrial cytopainter and seeded together before HGF (100 ng/ml) (24 h) treatment. Real-time acquisition of merged DIC and fluorescent images was captured at 10-min intervals. DiO-labelled vesicle (green) transfer was observed, and bidirectional transfer is inferred through the presence of mitochondria (red) in the DiO (green) labelled cell below. Scale bar, 20 μm.Download video

Video 5 Time-lapse movie of two DiO-labelled vesicles transferred along an HGF-induced TNT. A549 cells were treated with HGF (100 ng/ml) for 24 h and loaded with DiO before undertaking live-cell imaging. Real-time acquisition of merged DIC and fluorescent images was captured at 10-min intervals. The time-lapse movie shows unidirectional transfer of two DiO-labelled vesicles (green) along a TNT. Scale bar, 20 μm.Download video

### C-Met receptor regulates TNT formation

**Figure 4.**
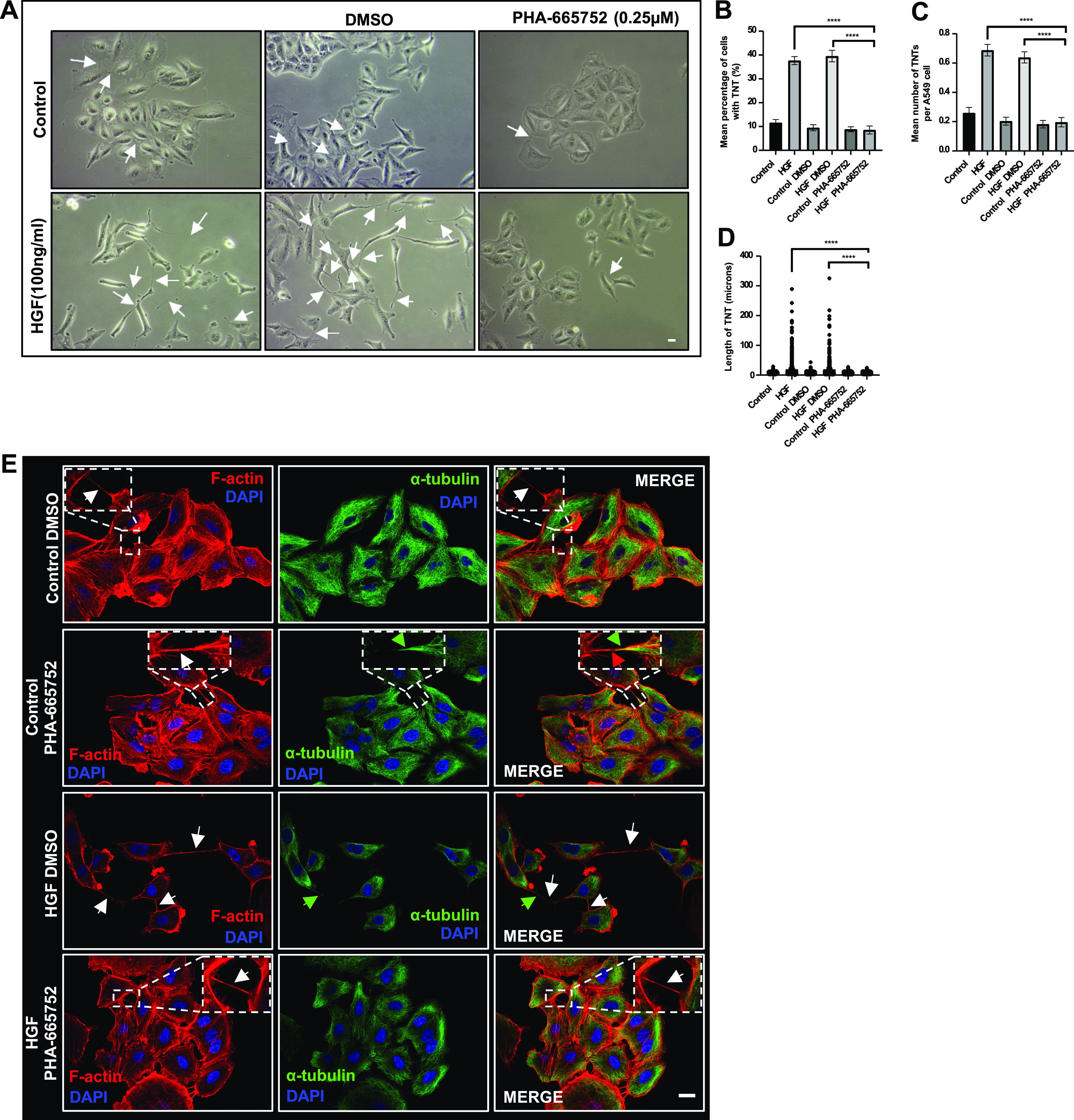
C-Met receptor mediates TNT formation in A549 cells. **(A)** Representative phase contrast images of control or HGF-treated cells (24 h) (100 ng/ml) pre-treated with PHA-665752 (0.25 μM) or DMSO. A decrease in TNTs (white arrows) in both control conditions (DMSO and PHA-665752) was observed. Phase contrast images were captured using 10x objective on an inverted microscope. Scale bar, 10 μm. **(B, C, D)** There was a significant decrease in HGF-induced TNTs in mean (B), number (C), and length (D) of TNTs in the presence of PHA-665752 compared with HGF DMSO–treated cells. **(E)** Representative immunofluorescence confocal images of control or HGF-treated cells (24 h) (100 ng/ml) pre-treated with PHA-665752 (0.25 μM) or DMSO and labelled with F-actin (red) and α-tubulin (green). There were fewer F-actin-positive HGF-induced TNTs (white arrows) observed in the presence of PHA-665752 compared with DMSO. The dotted region denotes a zoomed area. Scale bar, 20 μm. Values are expressed as mean ± SEM, n = 3, with at least 900 cells analysed per condition. ****P < 0.0001 when comparing HGF + PHA-665752-treated cells with either HGF or HGF + DMSO-treated cells.

**Figure S2.**
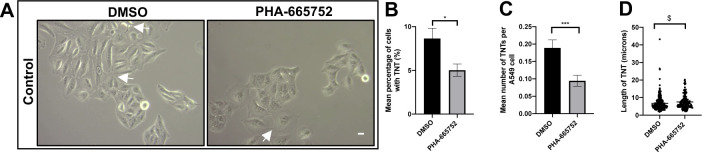
C-Met receptor inhibition decreses control TNTs. **(A)** Representative white light images of A549 cells treated with PHA-665752(1 μM) or DMSO. White arrow denotes a TNT structure. White light images were captured using 10x objective on an inverted microscope. Scale bar, 10 μm. **(B, C)** There was a significant decrease in the mean percentage (B) and mean number (C) of TNTs. **(D)** A significant increase in TNT length was observed in the PHA-6665752-treated cells compared with DMSO. Values are expressed as mean ± SEM, n = 3 with at least 400 cells analysed per condition. *P < 0.05 and ***P < 0.001 when compared with respective DMSO controls. $*P* < 0.05 when PHA-665752 TNT length was compared with DMSO control.

To further investigate the role of HGF in TNT formation, a c-Met inhibitor (PHA-665752; 0.25 μM) was used. HGF treatment in the presence of the c-Met inhibitor showed a reduction in TNTs compared with the HGF DMSO–treated cells (shown by white arrows) (Fig 4A) and exhibited a cuboidal epithelial cell morphology similar to control DMSO cells on phase contrast images. Quantification of TNTs in the phase contrast images demonstrated a significant decrease (n = 3, ****P < 0.0001) in the mean percentage of cells with TNTs (Fig 4B), mean number of TNTs per cell (Fig 4C), and TNT length (Fig 4D) of HGF-induced TNTs in the presence of the c-Met inhibitor compared with HGF DMSO–treated cells. Representative confocal images demonstrated expression of Phalloidin/F-actin (red) throughout the TNT length with α-tubulin (green) expressed in the thicker regions of the TNT (Fig 4E). Moreover, control cells treated with a higher concentration of the c-Met inhibitor (1 μM), showed a reduction in TNTs when compared with control DMSO-treated cells (shown by white arrows) (Fig S2A). Quantification of TNTs revealed a significant decrease in the mean percentage of TNTs (n = 3, *P < 0.05) and mean number of TNTs per cell (n = 3, ***P < 0.001) compared with control DMSO-treated cells (Fig S2B and C), while a significant increase in TNT length (n = 3, *$P* < 0.05) was observed in the presence of the c-Met inhibitor compared with control DMSO-treated cells (Fig S2D).

### β1-integrin receptor plays a role in HGF-induced TNT formation

**Figure 5.**
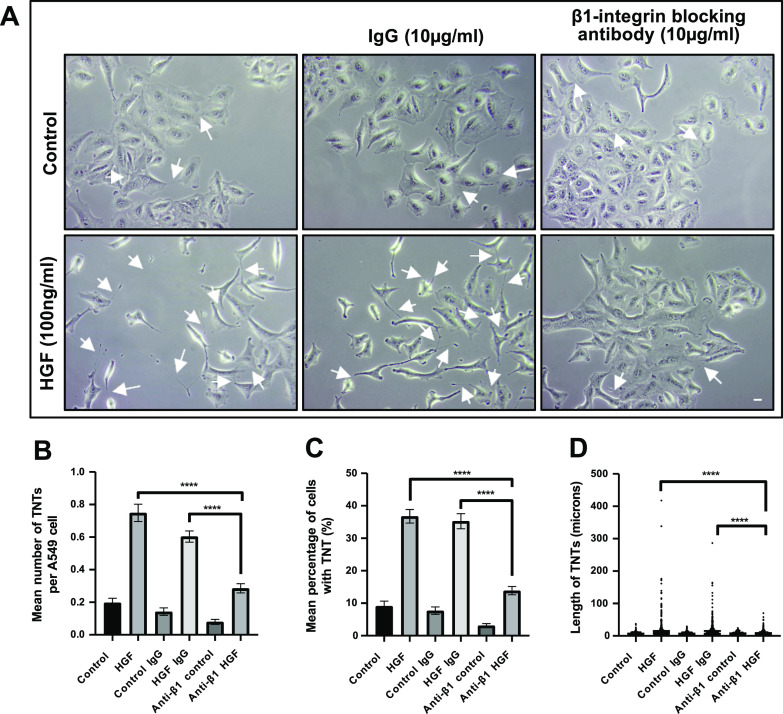
β1-integrin mediates the HGF-induced TNT formation in A549 cells. A549 cells were pre-treated with either IgG or a functionally blocking an anti–β1-integrin blocking antibody (10 μg/ml) for 30 min before HGF (100 ng/ml) treatment for 24 h. **(A)** Representative phase contrast images show a decrease in TNTs (white arrows) and colonies of epithelial cell morphology observed in the presence of β1-integrin antibody when A549 cells were treated with HGF compared with its IgG. Phase contrast images were captured using 10x objective on an inverted microscope. Scale bar, 10 μm. **(B, C, D)** The phase contrast images were quantified for TNTs for the mean percentage (B), mean number (C), and length (D) of TNTs. **(B, C, D)** There was a significant decrease in HGF-induced TNTs quantified in the presence of the β1-integrin antibody compared with HGF IgG (B, C, D). Values are expressed as mean ± SEM, n = 3, with at least 600 cells analysed per condition. ****P < 0.0001 when comparing between anti–β1-integrin antibody + HGF-treated cells with either HGF or HGF + IgG-treated cells.

To determine whether the β1-integrin was implicated in TNT formation, a functionally blocking β1-integrin antibody was used to determine if it could regulate the HGF-induced TNT formation in A549 cells. Representative phase contrast images showed a decrease in HGF-induced TNTs in the presence of the β1-integrin blocking antibody, compared with the HGF IgG-treated cells (Fig 5A). Further quantification of the phase contrast images demonstrated a significant decrease (n = 3, ****P < 0.0001) in mean percentage of cells with TNTs (Fig 5B), the mean number of TNT per cell (Fig 5C), and TNT length (Fig 5D) in HGF-treated cells in the presence of the β1-integrin blocking antibody, compared with the HGF IgG-treated cells. These results suggest a role for both β1-integrin and HGF/c-Met in regulating TNT formation in A549 cells.

### C-Met, β1-integrin, and paxillin are novel components of TNTs

**Figure 6.**
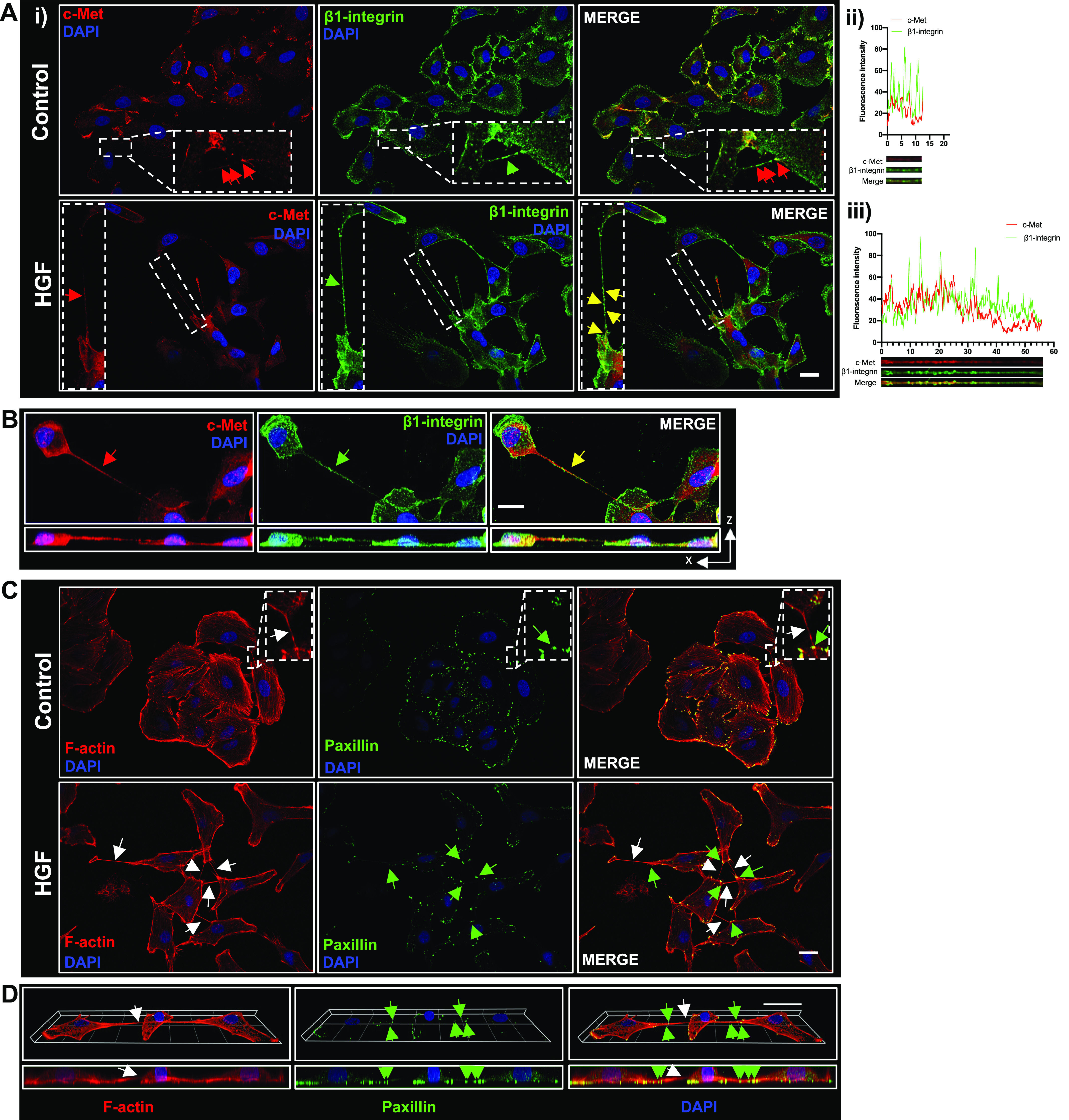
TNTs express novel components c-Met, β1-integrin, and paxillin. **(A)** (i) Representative confocal images for immunofluorescent labelled TNTs with c-Met (red) and β1-integrin (green) in control and HGF-treated A549 cells. C-Met and β1-integrin co-localised along the TNTs (yellow arrow). **(A)** (ii, iii) The line scan shows the fluorescence intensity profiles of the region of interests (as denoted by the dotted region). **(A)** Partial co-localisation of c-Met and (red) and β1-integrin (green) was observed in both control (A) (ii) and HGF-induced TNTs (A) (iii). **(B)** An example of a non-adherent TNT expressing c-Met (red) and β1-integrin (green). The aerial and orthogonal xz projection image was obtained using ZEN lite software from 39 sections of z-stacked images (with Z step of 0.27 μm). **(C)** Representative confocal images for immunofluorescent labelled TNTs with paxillin (green) and F-actin (red) in control and HGF-treated A549 cells. Paxillin is expressed at the protrusion site of the control and HGF-induced TNTs (green arrows). The white arrows denote TNT structures, and the dotted regions denote a zoomed area. **(D)** An example of a non-adherent F-actin–labelled TNT (white arrow) with paxillin (green arrows) expressed at the protrusion site at both ends of the TNT. The 3D reconstruction was acquired using the ZEN lite software from 39 sections of z-stacked images (with Z step of 0.27 μm). The maximum xz projection image below demonstrates the TNT hovering above the surface of the substratum. Scale bar, 20 μm.

After our findings demonstrating that c-Met and β1-integrin regulate the HGF-induced TNT formation, we next determined the localisation of c-Met and β1-integrin receptors in control and HGF-induced TNTs via immunofluorescent labelling and confocal microscopy. In control cells, c-Met and β1-integrin localised at the cell membrane; however, HGF treatment induced cytoplasmic and nuclear internalisation of c-Met as previously observed (Ménard et al, 2014). Analysis of c-Met and β1-integrin distribution showed that both proteins were present in all TNTs, i.e., control and HGF-induced TNTs. β1-integrin (green arrow) was expressed throughout the entire length of TNTs in 100% of TNTs, while c-Met (red arrow) was expressed throughout the length in 69% of TNTs. C-Met expression was localised at regions proximal to cells in 31% of TNTs (Fig 6Ai). Furthermore, c-Met and β1-integrin co-localised in TNTs in control and HGF-treated cells (yellow arrow) (Fig 6Ai). Representative images and line scans of both control and HGF-treated TNTs showed evidence of distinct expression of c-Met and/or β1-integrin on vesicle-like structures along the TNT (Fig 6Aii and iii). Analysis of the co-localisation between c-Met and β1-integrin in TNTs revealed a Pearson’s correlation coefficient value of 0.36 ± 0.02 for control TNTs and 0.34 ± 0.01 for HGF-induced TNTs, thus confirming c-Met and β1-integrin partial co-localisation. The Mander’s coefficient analysis also revealed strong c-Met and β1-integrin overlap with M1 (c-Met overlapping with β1-integrin) values of 0.86 ± 0.02 and 0.82 ± 0.02 for control and HGF-induced TNTs respectively, while M2 (β1-integrin overlapping with c-Met) showed a value of 0.69 ± 0.02 and 0.66 ± 0.01 for control and HGF-induced TNTs respectively.

To assess the non-adherence of the c-Met and β1-integrin–labelled TNTs, confocal z-stack acquisition was obtained to visualise an aerial and xz orthogonal view of the TNT (Fig 6B). The xz orthogonal view shows a c-Met and β1-integrin–positive TNT non-adherent to the substratum.

C-Met and β1-integrin activation have been shown to increase the tyrosine phosphorylation and thus association of paxillin via FAK, acting as a point in downstream convergence between HGF/c-Met and β1-integrin signalling (Schaller et al, 1995; Liu et al, 2002; Ishibe et al, 2003; Crowe & Ohannessian, 2004). Therefore, paxillin localisation was assessed through immunofluorescent labelling of control and HGF-induced TNTs. Paxillin (green arrows) was localised at the protrusion site of the F-actin-positive TNTs in both control and HGF-treated cells (Fig 6C). Non-adherence of the TNTs was also demonstrated through xz orthogonal view of paxillin-expressing TNTs (Fig 6D).

### Arp2/3 complex, MAPK, and PI3K pathways mediate TNT formation in A549 cells

**Figure 7.**
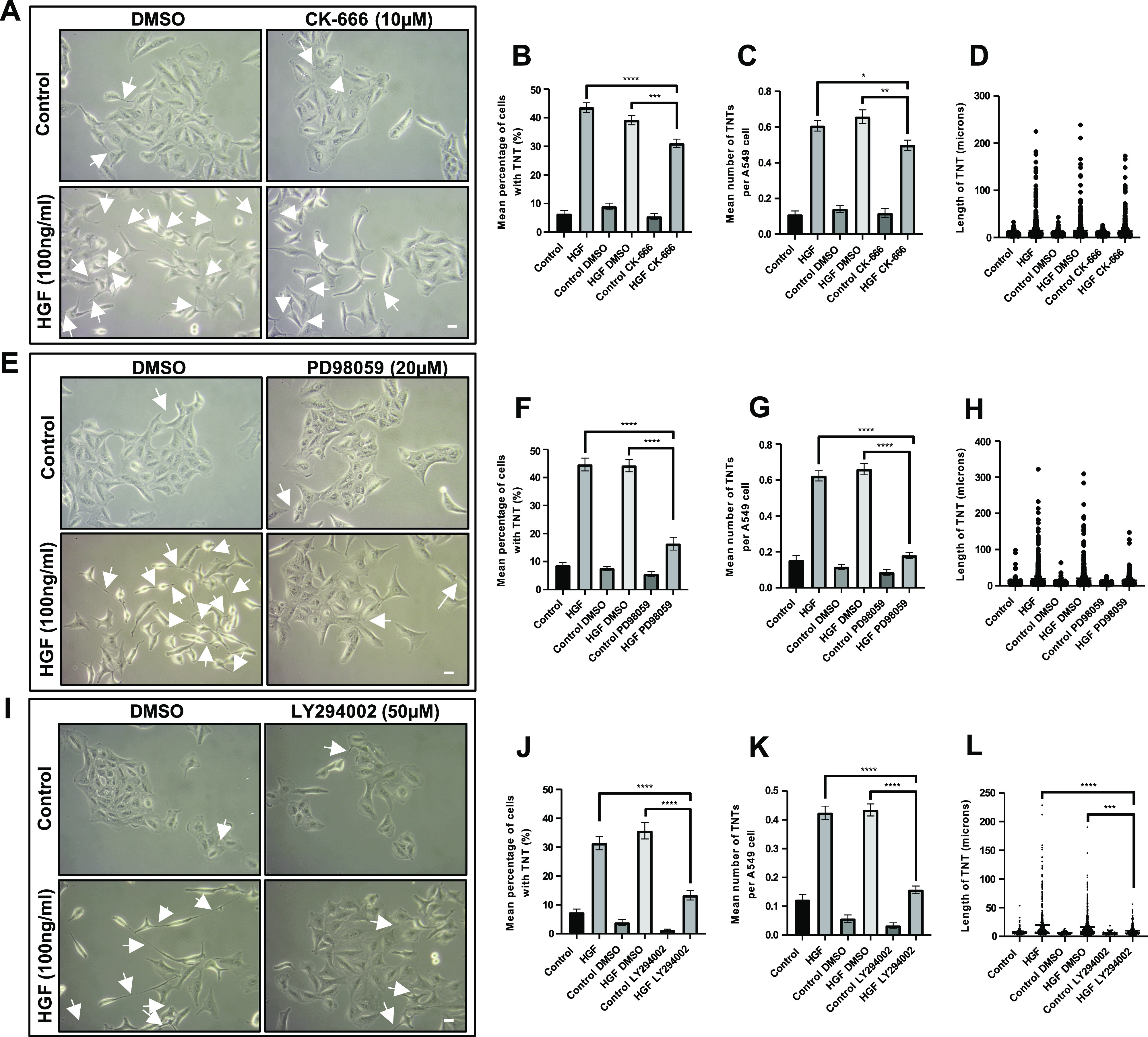
HGF induces TNTs via the MAPK, PI3K and Arp2/3 complex pathways. A549 cells were pre-treated with inhibitors associated with the HGF/c-Met downstream pathways or vehicle control for 30 min before HGF treatment (100 ng/ml) (24 h). **(A)** Representative phase contrast images of control or HGF-treated cells in the presence of CK-666 (10 μM) or DMSO. White arrow denotes a TNT structure. Phase contrast images were captured using 10x objective on an inverted microscope. Scale bar, 10 μm. **(B, C, D)** CK-666 significantly decreased the mean (B) and number (C) but not the length (D) of HGF-induced TNTs compared with HGF DMSO–treated cells. **(E)** Representative phase contrast images of control or HGF-treated cells in the presence of PD98059 (20 μM) or DMSO. **(F, G, H)** PD98059 significantly decreased the mean (F) and number (G) but not the length (H) of HGF-induced TNTs compared with HGF DMSO–treated cells. **(I)** Representative phase contrast images of control or HGF-treated cells in the presence of LY294002 (50 μM) or DMSO. **(J, K, L)** LY294002 significantly decreased the mean (J), number (K), and length (L) of HGF-induced TNTs compared with HGF DMSO–treated cells. Values expressed as mean ± SEM, n = 3 with at least 900 cells analysed per condition. *P < 0.05, **P < 0.01, ***P < 0.001, and ****P < 0.0001 when comparing between HGF + inhibitor-treated cells and either HGF or HGF + DMSO-treated cells.

**Figure S3.**
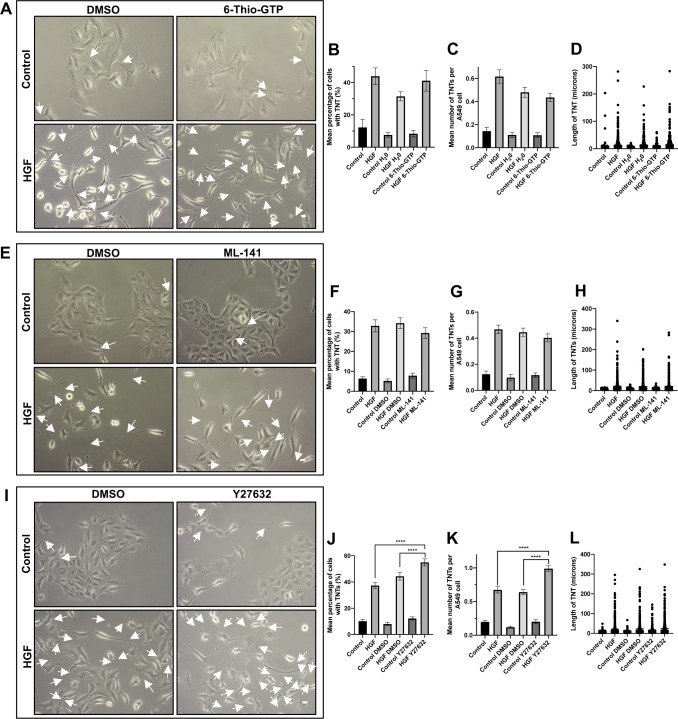
HGF does not induce TNTs via the ROCK, Rac1, and CDC42 pathways. A549 cells were pre-treated with inhibitors associated with the HGF/c-Met downstream pathways or vehicle control for 30 min before HGF treatment (100 ng/ml) (24 h). **(A)** Representative white light images of control or HGF-treated cells in the presence of 6-Thio-GTP (10 μM) or DMSO. **(B, C, D)** In the presence of 6-Thio-GTP, there was no significant difference in mean percentage (B), number (C), and length (D) of TNTs compared with HGF H_2_0-treated cells. White arrow denotes a TNT structure. White light images were captured using 10x objective on an inverted microscope. Scale bar, 10 μm. **(E)** Representative white light images of control or HGF-treated cells in the presence of ML-141 (10 μM) or DMSO. **(F, G, H)** In the presence of ML-141, there was no significant difference in mean percentage (F), number (G), and length (H) of TNTs compared with HGF DMSO–treated cells. **(I)** Representative white light images of control or HGF-treated cells in the presence of Y27632 (5 μM) or DMSO. **(J, K, L)** The presence of Y27632 induced a significant increase in mean percentage (J), number (K), and length (L) of HGF-induced TNTs compared with DMSO HGF-treated cells. Values are expressed as mean ± SEM, n = 3 with at least 500 cells analysed per condition. ****P < 0.0001 when comparing HGF Y27632-treated cells with either HGF treatment or HGF DMSO–treated cells.

To determine the downstream pathways involved in HGF-induced TNT formation, inhibitors of different cell signalling pathways were used. The Arp2/3 complex is activated downstream of CDC42/N-WASP and Rac1/WAVE pathways and has been shown to organise actin filaments into branched networks and are implicated in TNT formation (Hanna et al, 2017). Representative phase contrast images showed a decrease in HGF-induced TNTs (white arrows) in the presence of the Arp2/3 complex inhibitor (CK-666; 10 μM) when compared with the HGF DMSO–treated cells (Fig 7A). The presence of the Arp2/3 complex inhibitor induced a partial inhibition of HGF-induced TNTs (Fig 7A). There was a significant reduction of HGF-induced TNTs in mean percentage of cells with TNTs (Fig 7B) (n = 3, ***P < 0.001) and the mean number of TNTs per cell (Fig 7C) (n = 3, **P < 0.01) when compared with the HGF DMSO–treated cells, but had no effect on the TNT length (Fig 7D). Further examination of the Rho-A, Rac1, and CDC42 downstream signalling pathway demonstrated no effect of the Rac1 inhibitor (6-Thio-GTP; 10 μM) (Fig S3A–D) or the CDC42 inhibitor (ML-141; 10 μM) (Supplementary Fig S3E–H) on HGF-induced TNT formation when compared with the HGF DMSO–treated cells. The ROCK inhibitor (Y27632; 5 μM) (Fig S3I–L) significantly (n = 3, ****P < 0.0001) increased the mean percentage of TNTs (Fig S3J), mean number of TNTs per cell (Fig S3K), and length (Fig S3L) of HGF-induced TNTs compared with the HGF DMSO–treated cells.

MAPK (PD98059; 20 μM) inhibition had no effect on control cell morphology or TNT formation and was comparable to DMSO control cells (Fig 7E). However, very few TNTs were visible in control cells when they were cultured in the presence of the MAPK inhibitor compared with HGF DMSO–treated cells. Quantification of phase contrast images showed the MAPK inhibitor significantly decreased HGF-induced TNT formation (n = 3, ****P < 0.0001) in the mean percentage of cells with TNTs (Fig 7F) and mean number of TNT per cell (Fig 7G) compared with the HGF DMSO–treated cells, but had no effect on the TNT length (Fig 7H).

PI3K (LY294002; 50 μM) inhibition had no effect on control cell morphology or TNT formation and was comparable to DMSO control cells (Fig 7I). However, very few TNTs were visible in control cells when they were cultured with the PI3K inhibitor compared with HGF DMSO–treated cells. Quantification of images showed that the PI3K inhibitor significantly decreased HGF-induced TNTs (n = 3, ****P < 0.0001) in the mean percentage (Fig 7J) and mean number of TNT per cell (Fig 7K) compared with the HGF DMSO–treated cells. The PI3K inhibitor had no effect on the HGF-induced TNT length when compared with the HGF DMSO–treated cells (Fig 7L).

**Figure S4.**
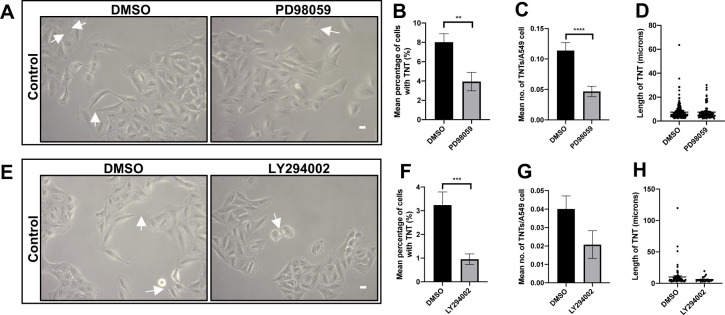
MAPK and PI3K inhibition decreases control TNTs. **(A)** Representative white light images of A549 cells treated with PD98059 (40 μM) or DMSO. White arrow denotes a TNT structure. White light images were captured using 10x objective on an inverted microscope. Scale bar, 10 μm. **(B, C, D)** There was a significant decrease in the mean percentage (B) and mean number (C) but not in length (D) of TNTs. **(E)** Representative white light images of A549 cells treated with LY294002 (100 μM) or DMSO. **(F, G, H)** There was a significant decrease in the mean percentage (F) but not in mean number (G) and length (H) of TNTs. Values are expressed as mean ± SEM, n = 3 with at least 400 cells analysed per condition. **P < 0.01, ***P < 0.001, and ****P < 0.0001 when compared with respective DMSO controls.

Supplementary data (Fig S4A–D) showed that PD98059, at a higher concentration of 40 μM, significantly decreased the mean percentage of cells with TNTs (n = 3, **P < 0.01) (Fig S4B) and mean number of TNTs per cell (n = 3, ****P < 0.0001) (Fig S4C) when compared with control DMSO-treated cells. No significance was observed in TNT length between the PD98059-treated and control DMSO-treated cells (Fig S4D). A higher concentration of LY294002 (100 μM) (Fig S4E–H) showed a significant decrease (n = 3, ***P < 0.001) in the mean percentage of TNTs when compared with control DMSO-treated cells (Fig S4F). No significance was observed in mean number of TNT per cell (Fig S4G) and TNT length (Fig S4H) between the PD98059-treated and control DMSO-treated cells.

### Paxillin regulates HGF-induced TNT formation

**Figure 8.**
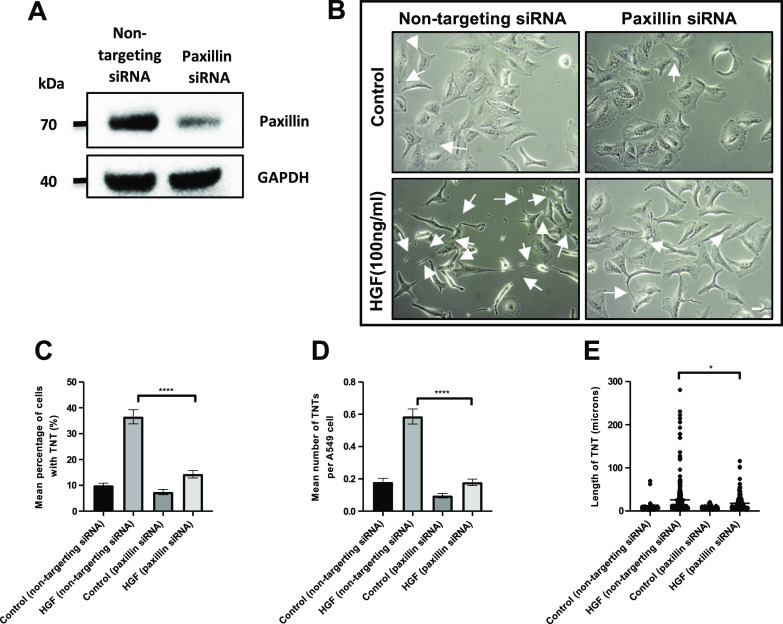
Paxillin regulates HGF-induced TNT formation. **(A)** Western blot shows knockdown of paxillin in A549 cells after 48 h transfection with paxillin siRNA. **(B)** Representative phase contrast images of A549 cells transfected with paxillin siRNA or non-targeting siRNA for 48 h before 24 h HGF treatment (100 ng/ml). **(C, D, E)** There was a significant decrease in mean percentage (C), number (D), and length (E) of TNTs between HGF-treated cells transfected with paxillin siRNA compared with cells transfected with non-targeting siRNA. Values are expressed as mean ± SEM, n = 3 with at least 500 cells analysed per condition. *P < 0.05, and ****P < 0.0001 when comparing between HGF-treated cells transfected with siRNA paxillin and HGF-treated cells transfected with a non-targeting siRNA.

**Figure S5.**
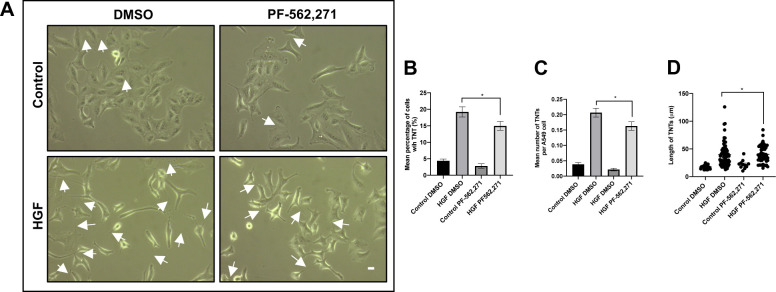
FAK inhibitor decreases HGF-induced TNTs. **(A)** Representative white light images of A549 cells treated with PF-562,271 (1 μM) or DMSO. White arrow denotes a TNT structure. White light images were captured using 10x objective on an inverted microscope. Scale bar, 10 μm. **(B, C, D)** There was a significant decrease in the mean percentage (B) and mean number (C) but not in length (D) of TNTs between HGF DMSO and HGF PF-562,271 TNTs. Values are expressed as mean ± SEM, n = 3, with at least 100 cells analysed per condition. *P < 0.05 DMSO controls.

The next step was to determine the role of paxillin in TNT formation, through transfection of A549 cells with siRNA to paxillin to achieve knockdown. Western blot analysis confirmed significant downregulation of paxillin expression in the A549 cells transfected with the siRNA for paxillin compared with a non-targeting siRNA (Fig 8A). Moreover, phase contrast images revealed a significant decrease in HGF-induced TNTs when A549 cells were transfected with a siRNA for paxillin, whereas the cells transfected with the non-targeting siRNA displayed elongated morphology and the usual increase in HGF-induced TNTs (Fig 8B). There was a significant decrease in the mean percentage of TNTs (n = 3, ****P < 0.0001) (Fig 8C), the mean number of TNTs per cell (n = 3, ****P < 0.0001) (Fig 8D), and mean TNT length (n = 3, *P < 0.05) (Fig 8E) of HGF-induced TNTs when transfected with the paxillin siRNA compared with the HGF-treated cells transfected with the non-targeting siRNA. Furthermore, inhibition of FAK (Fig S5A–D), which acts as a signalling scaffold with paxillin, resulted in a significant reduction (n = 3, *P < 0.05) in the mean percentage (Fig S5B), mean number of TNTs per cell (Fig S5C), and mean length of HGF-induced TNTs (Fig S5D) when compared with HGF DMSO–treated cells.

## Discussion

This work highlights a novel role for the HGF/c-Met/β1-integrin signalling axis in inducing TNT formation in A549 cells. HGF induced TNTs in a concentration- and time-dependent manner. HGF-induced TNTs demonstrated characteristic TNT non-adherence to the culture substratum, expressed F-actin and α-tubulin, and transported mitochondria and lipid vesicles. C-Met, β1-integrin, and paxillin were identified as novel components of TNTs. C-Met and β1-integrin co-localised along the TNT length, and paxillin was identified at the TNT protrusion site. Inhibition of the c-Met receptor or blocking β1-integrin with a functionally blocking antibody abrogated HGF-induced TNTs. Pharmacological inhibition of downstream pathways PI3K, MAPK, and the Arp2/3 complex also suppressed HGF-induced TNT formation. Control TNTs were also regulated by the PI3K and MAPK signalling pathways. Furthermore, knockdown of paxillin, through siRNA transfection, inhibited HGF-induced TNTs, suggesting a central role for paxillin in β1-integrin and HGF/c-Met interactions in TNT formation.

HGF induced TNTs in a time- and concentration-dependent manner. The concentrations required to elicit this response were the same concentration ranges that induce other HGF responses such as cell growth (Puri & Salgia, 2008), migration (McBain et al, 2003; Kermorgant et al, 2004; González et al, 2017), and invasion (Kermorgant et al, 2001; Syed et al, 2011). Furthermore, the shape of the HGF concentration-response curve was also characteristic of HGF and other growth factors, displaying a dome-shaped curve at higher concentrations (To et al, 2002). These findings suggest that HGF exerts a multitude of effects on cells simultaneously. Indeed, in our time-lapse videos, we observed cell division, migration alongside TNT formation, indicating the complex biology of HGF. The length of TNTs induced by HGF reached up to 400 μm in our study. These longer lengths are more typical of tumour microtubes that are present in the aggressive cancer glioblastoma, but TMs are thicker than TNTs (Roehlecke & Schmidt, 2020). Taken together, this suggests that HGF is a strong driver of TNT formation.

HGF-induced TNTs were non-adherent to the substratum and also travelled over neighbouring cells to connect with distant cells; this has also been previously observed in other studies (Lou et al, 2012; Desir et al, 2016). HGF-induced TNTs expressed F-actin and α-tubulin, which are characteristic of the thin and thick TNTs observed in A549 cells (Wang et al, 2012, 2021; Kumar et al, 2017; Dubois et al, 2018). We also demonstrated the capability of HGF-induced TNTs to traffic lipid vesicles and mitochondria in both a unidirectional and bidirectional manner. This type of uni/bidirectional transfer has been seen in other cells, including mesothelioma cells (Lou et al, 2012), B cells (Osteikoetxea-Molnár et al, 2016), microglia (Scheiblich et al, 2021), and myeloid leukemia cells (Kolba et al, 2019). Several studies have alluded to TNT-mediated mitochondria transfer as part of a survival mechanism (Caicedo et al, 2015; Wang & Gerdes, 2015; Lin et al, 2019). Another study showed A549 cells were able to transfer healthy mitochondria to other cells suffering drug-induced mitochondrial loss, thus restoring aerobic metabolism and delaying apoptosis (Spees et al, 2006). Therefore, in our study the HGF-induced mitochondria transfer may be a mechanism to rescue apoptotic cells within the TME. Furthermore, although we have identified lipid vesicles and mitochondria as being trafficked, we do not exclude the trafficking of other organelles such as lysosomes (Abounit et al, 2016) or even oncogenic microRNAs (Thayanithy et al, 2014), which has been previously reported. Indeed, the potential for HGF-induced TNT trafficking could also have implications for chemoresistance that could involve movement of oncogenes, like KRAS (Desir et al, 2019) or potential drug efflux transporters (Desir et al, 2018).

Identification of HGF as an inducer of TNTs resulted in the discovery of three novel components of TNTs involved in HGF signalling, namely paxillin, c-Met, and β1-integrin. There was distinct localisation of each of these components, with paxillin restricted to the TNT protrusion site and β1-integrin and c-Met present along the length of TNTs. C-Met can phosphorylate paxillin, which alters the cytoskeleton, causing migration (Ma et al, 2003). This work suggests that paxillin’s role extends to facilitating TNT formation as it localised at the protrusion site and had a distinctly different localisation in control cells, which had very few TNTs; future work will delineate how paxillin could achieve this. Both β1-integrin and c-Met were present in all TNTs and co-localised, which has been shown before in different cellular responses such as anchorage-dependent survival (Barrow-McGee et al, 2016), migration, metastasis (Mitra et al, 2011), and chemotherapy resistance (Jahangiri et al, 2017). TNTs also contained vesicle-like structures expressing both β1-integrin and c-Met, suggesting that they could be packaged together. However, there were examples where distinct vesicles either containing c-Met or β1-integrin were also distinguished. Literature on how vesicles are packaged, mobilised, and targeted in TNTs is lacking but will be an important aspect for future work.

Either blocking β1-integrin or inhibiting the c-Met receptor abrogated HGF-induced TNTs, suggesting a crosstalk between these two pathways. Previous studies have shown β1-integrin and c-Met can internalise together to aid NSCLC cell survival (Barrow-McGee et al, 2016). Another study showed siRNA knockdown of β1-integrin in conjunction with c-Met inhibition abrogated HGF-induced cell growth in NSCLC cell lines and demonstrated the ability of β1-integrin to phosphorylate the c-Met receptor (Ju & Zhou, 2013). The β1-integrin/c-Met complex has also been implicated in the progression of breast cancer metastasis (Lau et al, 2021). Our findings show c-Met receptor inhibition reduced the control TNTs, indicating the existence of an autocrine signalling loop, as previously reported (Masuya et al, 2004; Nakamura et al, 2007), suggesting a fundamental role in c-Met signalling in not only growth, metastasis, and survival but also in TNT formation.

Another study has shown similar results when interrogating the role of β1-integrin in TNT formation of B lymphocytes, highlighting the importance of β1-integrin, but in contrast, finding that both the α5 integrin and the β1-integrin subunits are required for TNT formation (Osteikoetxea-Molnár et al, 2016). Extracellular matrix such as fibronectin and collagen I can also induce TNTs (Franchi et al, 2020; Wang et al, 2021), again indicating a role for different integrin subunits in TNT formation. We also showed FAK inhibition caused a reduction in HGF-induced TNTs. β1-integrin can phosphorylate c-Met and activate FAK and Akt in other NSCLC cells (Ju & Zhou, 2013). FAK in focal adhesion sites can also upregulate matrix metalloproteinases (MMPs), which promotes TNT formation in squamous cell carcinoma (Sáenz-de-Santa-María et al, 2017). HGF can also upregulate MMPs (González et al, 2017), so it may play a role in the HGF-induced TNT formation observed in this study. MMPs are important to consider as they alter ECM composition, thus changing the cell-integrin expression/ligation (Stamenkovic, 2003). The difference in these findings suggests a distinct regulatory role of integrin subunits in determining cell-specific TNT formation. Integrins also localise at focal adhesion sites and can help regulate dynamics of the actin cytoskeleton (Case & Waterman, 2015), which is important in TNT formation. Overall, this suggests the dynamic changes occurring in cellular adhesion and ECM remodeling underly the cytoskeletal reorganisation required in TNT formation.

In addition to c-Met and β1-integrin, siRNA knockdown of paxillin resulted in abrogation of HGF-induced TNTs. Paxillin has been shown to play a role in the β1-integrin–mediated migration with paxillin localising at the protrusion site in the early stages of focal adhesion formation (Laukaitis et al, 2001). Paxillin, as a scaffold protein, is known to associate with β1-integrin in squamous cell carcinoma (Crowe & Ohannessian, 2004) and in Schwann cells (Chen et al, 2000) with its phosphorylation mediated by the c-Met receptor (Laukaitis et al, 2001). Amongst the various mutationally upregulated EMT-inducing cytokines present in the TME, only EGF and its receptor signalling have been widely studied in TNT formation (Carter et al, 2019; Hanna et al, 2019; Cole et al, 2021).

In this study, CDC42 and Rac1 inhibition showed no effect on the HGF-induced TNT formation (despite higher concentrations being used, data not shown); this is in contrast to other studies performed in macrophages (Hanna et al, 2017). Despite TNTs being actin-rich structures, there is increasing evidence to suggest that the Rho GTPase pathways play a more complex role in TNT formation, dependent on the different cell types. Although CDC42 activation has been proposed in the formation of TNTs in immune cells (Arkwright et al, 2010; Hanna et al, 2017), CDC42 inhibition only minimally decreased the LST1-induced TNT formation (Schiller et al, 2013). Rac1 has been proposed to sustain existing TNTs instead of playing a role in their formation (Hanna et al, 2017). As the molecular mechanisms underlying TNT formation differ between cell types, our study suggests the Rho GTPases CDC42 and Rac1 are not involved in the HGF-induced TNT formation in A549 cells. Further investigations, via siRNA knockdown/CRISPR-knockout studies, would be required to confirm whether CDC42 and Rac1 are not involved in the HGF-mediated TNT formation. We also observed ROCK inhibition promoted an increase in HGF-induced TNTs. Similarly, other studies in mesothelioma and microglia cells also showed an increase in TNT formation in the presence of the same ROCK inhibitor (Scheiblich et al, 2021; Jana et al, 2022). In microglia cells, the increase in TNT formation was due to alterations in myosin II-mediated F-actin regulation (Scheiblich et al, 2021); however, this mechanism has not been investigated in A549 cells.

MAPK and PI3K inhibition also significantly decreased both basal and HGF-induced TNTs in our study; thus, both signalling pathways play a role in TNT formation of A549 cells. Although studies continue to unravel the mechanisms involved in TNT formation, the mechanisms and pathways involved in NSCLC TNT formation remain poorly defined. The MAPK pathway has been implicated in TNT formation in ovarian cancer cells and in astrocytes (Zhu et al, 2005; Cole et al, 2021). Interestingly, in ovarian cancer cells, the PI3K inhibitor did not inhibit TNT formation, whereas inhibition of the MAPK pathway decreased the number of TNTs (Cole et al, 2021). However, the PI3K pathway has been shown to be involved in TNT formation in prostate cancer cells (Kretschmer et al, 2019), in astrocytes (Wang et al, 2011), and in bladder cancer and kidney cells (D’Aloia et al, 2021), thus highlighting again the different molecular mechanisms underlying TNT formation in different cell types. Paxillin can also bind to various adaptor proteins and multiple downstream signalling effectors to activate the PI3K, MAPK, and Arp2/3 complex pathways (Lai et al, 2000; Ishibe et al, 2003; Crowe & Ohannessian, 2004), suggesting paxillin acts as an important link between the upstream and downstream players involved in the HGF/c-Met/β1-integrin cell signalling pathways in A549 cells. A partial decrease in TNTs was observed in the presence of the Arp2/3 complex inhibitor. Similarly in macrophages, partial inhibition of TNT formation was also observed (Hanna et al, 2017), thus highlighting the role of Arp2/3 complex in regulating TNT formation in different cell types. Furthermore, Arp2/3 complex is mainly activated upstream by CDC42-stimulated N-WASP or Rac1-stimulated WAVE. However, both CDC42 and Rac1 inhibitors did not decrease the HGF-induced TNTs. There may be crosstalk between both the CDC42 and Rac1 pathways in mediating the HGF-induced TNT formation; alternatively, Arp2/3 complex may be activated via an ERK-dependent phosphorylation of WAVE (Mendoza et al, 2011). This mechanism of Arp2/3 complex activation has been observed in EGFR activation with an increase in lamellipodia protrusions (Mendoza et al, 2011). Future work using siRNAs will help delineate this pathway.

**Figure 9.**
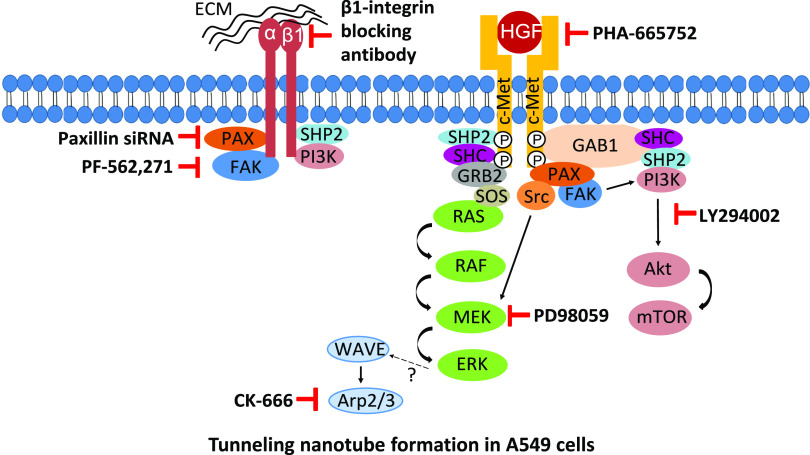
HGF/c-Met and β1-integrin signalling axis drives TNT formation in A549 cells. Paxillin is recruited after HGF/c-Met and β1-integrin activation and acts as a scaffold protein to activate Arp2/3 complex, PI3K, and MAPK downstream pathways. Arp2/3 complex-mediated TNT formation may occur via ERK-mediated phosphorylation of WAVE.

Understanding the complex underlying molecular mechanisms involved in TNT formation serves a wider implication in lung adenocarcinoma and for NSCLC therapy. This study demonstrates a novel role for HGF in inducing TNT formation in A549 cells while providing a proof of principle for the potential molecular mechanisms involved in TNT formation in NSCLC (Fig 9). This further highlights the importance of a personalised targeted approach in NSCLC treatment. Future work will focus on using different NSCLC cell lines and patient samples to determine the wider role of HGF in TNT formation. Further confirmation of the pathways identified through our pharmacological studies would also be required through either genetic CRISPR-knockout or siRNA knockdown. In vivo studies would also be needed to assess the occurrence of TNTs and c-Met expression in lung adenocarcinoma tissue. This would serve as the next step in targeting TNTs in NSCLC.

## Materials and Methods

### Reagents

Cytokine and inhibitors; purified recombinant human HGF was obtained from Peprotech. The c-Met receptor inhibitor (PHA-665752 ≤ 1 μM), MAPK pathway inhibitor (PD98059 ≤ 40 μM), Arp2/3 complex inhibitor (CK-666 ≤ 200 μM) and CDC42 inhibitor (ML-141 ≤ 20 μM) were obtained from Cayman Chemical. The ROCK pathway inhibitor (Y27632 ≤ 7.5 μM) and FAK inhibitor (PF-562,271; 1 μM) were purchased from Merck. The PI3K pathway inhibitor (LY294002 ≤ 100 μM) and the Rac1 inhibitor (6-Thio-GTP≤50 μM) were obtained from Selleck Chemicals and Jena Bioscience, respectively. The functionally blocking β1-integrin antibody (≤10 μg/ml) and its mouse IgG1 isotype were purchased from Abcam.

Immunofluorescence; primary and secondary antibodies, rabbit anti-Met, and rat anti-α-tubulin were obtained from Invitrogen. Mouse anti-paxillin was obtained from BD Bioscience. Mouse anti–β1-integrin was obtained from Abcam. Alexafluor-conjugated secondary antibodies (488, 568, and 647 nm), Rhodamine phalloidin, DiO’, and DiOC_18_(3) (3,3′-Dioctadecyloxacarbocyanine Perchlorate) were all purchased from Invitrogen. The mitochondrial staining kit, red fluorescence-cytopainter, was purchased from Abcam. Vectashield mounting medium was obtained from Vector Laboratories Ltd., and DAPI was obtained from Invitrogen.

### Cell culture

The A549 lung adenocarcinoma cell line was obtained from the American Type Culture Collection (ATCC). The cells were cultured in DMEM (Gibco), and supplementation of the media required 10% FBS, 200 mM L-glutamine, 10,000 U/ml penicillin, and 10,000 μg/ml streptomycin (Gibco). The cell lines were cultured and maintained at 37°C with CO_2_ level of 5% in a humidified atmosphere.

A549 cells were seeded at a density of 5 × 10^3^ cells/well in a 12-well plate in 10% FBS-supplemented DMEM media. The cells were left to grow into colonies for 72 h in a humidified atmosphere previously described before treatment with HGF in 2% FBS-supplemented DMEM media for 24 h. For inhibitor studies, A549 cells were pre-treated for 30 min with the inhibitors of pathways detailed above with their respective vehicle controls. After pre-treatment, HGF (100 ng/ml) in 2% FBS-supplemented media was added for 24 h before undertaking phase contrast imaging. The respective inhibitor concentrations had no effect on cell viability when assessed using alamar blue assay (data not shown).

### siRNA transfection

The non-targeting siRNA (QIAGEN) and paxillin siRNA (QIAGEN) were transfected with HiPerfect (QIAGEN) as previously described (Porter et al, 2020). 20 μM of siRNA/well was transfected on a six-well plate. After 48 h of transfection, cells were trypsinised, and a fraction of the cells were used to seed on a 12-well plate for HGF treatment previously described. The remainder of cells were used for Western blot processing to confirm paxillin knockdown. The following paxillin siRNA target sequence was used: ATCCAAAGGCAGAGAACCAAA.

### Immunofluorescence

#### Confocal immunofluorescence microscopy

A549 cells were seeded at a density of 7.5 × 10^3^ cells on No. 0 coverslips (Thermo Fisher Scientific) before treatment with HGF and its respective pathway inhibitors, as previously described. Cells were then fixed with 4% PFA for 10 min, permeabilised with 0.5% Triton-X for 3.5 min, and blocked with 3% BSA before overnight incubation at 4°C with primary antibodies (1:100) in 3% BSA. For immunolabelling of c-Met, cells were fixed with 100% methanol for 10 min, permeabilised with 0.1% Triton-X for 10 min, and blocked with 3% BSA before overnight incubation at 4°C with anti-c-Met primary antibody (1:100) in 3% BSA. Visualization of immunolabelled proteins required the respective combination of species-specific Alexafluor-conjugated secondary antibodies (488, 568, and 647 nm) (1:200), raised in donkey or goat, co-incubated with rhodamine phalloidin. Immunolabelling protocol for paxillin was followed as per Sastry et al (1999). Cells were then imaged with Zeiss LSM980-Airyscan confocal microscope using 40x 1.3 NA oil objective.

#### Live-cell trafficking of mitochondria and vesicles

To visualise vesicle and mitochondria transfer along the TNTs, HGF pre-treated cells on coverslips were preloaded with red mitochondrial cytopainter for 30 min following the manufacturer’s instruction and then washed three times with PBS. Separate cell populations were also preloaded with either DiO (5 μM) or red mitochondrial cytopainter for 30 min. Both cell populations were then seeded together at a 1:1 ratio and treated with HGF for 24 h. Coverslips were mounted on a ludin chamber and enclosed in a humidified atmosphere at 37°C with 5% CO_2_. Real-time acquisition of images was captured at 30 s–2 min intervals for a duration of 20 min–4 h. Live cells were imaged with Zeiss LSM980-Airyscan confocal microscope using 40x 1.3 NA oil objective or on a Zeiss Axio Observer 7 microscope using a 20x 0.5 NA objective.

### TNT image analysis

#### Phase contrast image analysis

Phase contrast images were acquired with a Zeiss Primovert inverted microscope and GXCAM3EY-5 camera at x10 magnification. At least 10 fields of view per condition were captured with at least 400 cells analysed per condition. Using ImageJ software, the total number of cells was counted, and the number of cells with TNTs was counted to obtain the mean percentage of A549 cells with TNTs for each field of view. The images were also analysed to obtain the counts for the number of TNT per cell. Individual TNT lengths were also measured in μm, from the narrow initiation point to the end of the visible TNT length.

#### Confocal image analysis

Confirmation of lack of adherence was observed through z-stack acquisitions of F-actin labelled TNTs visible on higher focal planes. Z-stack images were acquired at 0.27–1.2 μm intervals. The ZEN lite software was used to obtain the 3D reconstruction and orthogonal view.

### Western blot

Protein extraction was performed with 4x Laemmli’s Buffer, which comprised of 0.5 M Tris pH 6.8 (12.5%), Glycerol (10%), SDS (2%), Bromophenol Blue (0.08%), and 2-mercaptoethanol (5%) in water. Protein lysates were denatured at 95°C for 5 min before being loaded into a TruPAGE 4–20% precast gradient gel (Sigma-Aldrich) and undergoing SDS–PAGE at 120 V for 2 h. Separated proteins were subsequently transferred onto a PVDF membrane at 30 V for 3 h. Membranes were blocked with 5% milk/TBST at room temperature for 1 h before being incubated overnight at 4°C with paxillin (Abcam) and GAPDH (Cell Signaling Technology) primary antibodies diluted in 5% BSA/TBST (1:500 and 1:4,000, respectively). After three washes with TBST, membranes were incubated at room temperature for 2 h in anti-rabbit (1:2,000) (Sigma-Aldrich) and anti-mouse (1:4,000) (Sigma-Aldrich) secondary antibodies diluted in 5% milk/TBST (1:2,000 and 1:4,000, respectively). After incubation, the signals were detected by ECL detection reagents (Amersham).

### Statistical analysis

Experiments were conducted at least three times with at least 500 cells analysed per condition. The data were expressed as the mean ± SEM for log dose-response curves, line graphs, scatter plots, and histograms comparing control with inhibitor-treated/siRNA knockdown groups. Determination of significance was assessed appropriately with either one-way ANOVA and Tukey’s multiple comparisons or through unpaired *t* test using Graphpad Prism 8. All statistical analysis was significant when *P* < 0.05.

## Supplementary Material

Reviewer comments

## References

[bib1] Abounit S, Bousset L, Loria F, Zhu S, de Chaumont F, Pieri L, Olivo‐Marin JC, Melki R, Zurzolo C (2016) Tunneling nanotubes spread fibrillar α‐synuclein by intercellular trafficking of lysosomes. EMBO J 35: 2120–2138. 10.15252/embj.201593411 27550960 PMC5048354

[bib2] Ariazi J, Benowitz A, De Biasi V, Den Boer ML, Cherqui S, Cui H, Douillet N, Eugenin EA, Favre D, Goodman S, (2017) Tunneling nanotubes and gap junctions–their role in long-range intercellular communication during development, health, and disease conditions. Front Mol Neurosci 10: 333. 10.3389/fnmol.2017.00333 29089870 PMC5651011

[bib3] Arkwright PD, Luchetti F, Tour J, Roberts C, Ayub R, Morales AP, Rodríguez JJ, Gilmore A, Canonico B, Papa S, (2010) Fas stimulation of T lymphocytes promotes rapid intercellular exchange of death signals via membrane nanotubes. Cell Res 20: 72–88. 10.1038/cr.2009.112 19770844 PMC2822704

[bib4] Barrow-McGee R, Kishi N, Joffre C, Ménard L, Hervieu A, Bakhouche BA, Noval AJ, Mai A, Guzmán C, Robbez-Masson L, (2016) Beta 1-integrin–c-Met cooperation reveals an inside-in survival signalling on autophagy-related endomembranes. Nat Commun 7: 11942. 10.1038/ncomms11942 27336951 PMC4931016

[bib5] Caicedo A, Fritz V, Brondello J-M, Ayala M, Dennemont I, Abdellaoui N, de Fraipont F, Moisan A, Prouteau CA, Boukhaddaoui H, (2015) MitoCeption as a new tool to assess the effects of mesenchymal stem/stromal cell mitochondria on cancer cell metabolism and function. Sci Rep 5: 9073. 10.1038/srep09073 25766410 PMC4358056

[bib6] Carter KP, Hanna S, Genna A, Lewis D, Segall JE, Cox D (2019) Macrophages enhance 3D invasion in a breast cancer cell line by induction of tumor cell tunneling nanotubes. Cancer Rep 2: e1213. 10.1002/cnr2.1213 PMC7254960 32467880

[bib7] Case LB, Waterman CM (2015) Integration of actin dynamics and cell adhesion by a three-dimensional, mechanosensitive molecular clutch. Nat cell Biol 17: 955–963. 10.1038/ncb3191 26121555 PMC6300998

[bib8] Chen L-M, Bailey D, Fernandez-Valle C (2000) Association of β1 integrin with focal adhesion kinase and paxillin in differentiating Schwann cells. J Neurosci 20: 3776–3784. 10.1523/JNEUROSCI.20-10-03776.2000 10804218 PMC6772705

[bib9] Cole JM, Dahl R, Cowden Dahl KD (2021) MAPK signaling is required for generation of tunneling nanotube-like structures in ovarian cancer cells. Cancers 13: 274. 10.3390/cancers13020274 33450985 PMC7828401

[bib10] Corso S, Comoglio PM, Giordano S (2005) Cancer therapy: Can the challenge be MET? Trends Mol Med 11: 284–292. 10.1016/j.molmed.2005.04.005 15949770

[bib11] Crowe DL, Ohannessian A (2004) Recruitment of focal adhesion kinase and paxillin to β1 integrin promotes cancer cell migration via mitogen activated protein kinase activation. BMC cancer 4: 18. 10.1186/1471-2407-4-18 15132756 PMC416481

[bib12] Desir S, Dickson EL, Vogel RI, Thayanithy V, Wong P, Teoh D, Geller MA, Steer CJ, Subramanian S, Lou E (2016) Tunneling nanotube formation is stimulated by hypoxia in ovarian cancer cells. Oncotarget 7: 43150–43161. 10.18632/oncotarget.9504 27223082 PMC5190014

[bib13] Desir S, O’Hare P, Vogel RI, Sperduto W, Sarkari A, Dickson EL, Wong P, Nelson AC, Fong Y, Steer CJ, (2018) Chemotherapy-induced tunneling nanotubes mediate intercellular drug efflux in pancreatic cancer. Sci Rep 8: 9484. 10.1038/s41598-018-27649-x 29930346 PMC6013499

[bib14] Desir S, Wong P, Turbyville T, Chen D, Shetty M, Clark C, Zhai E, Romin Y, Manova-Todorova K, Starr TK, (2019) Intercellular transfer of oncogenic KRAS via tunneling nanotubes introduces intracellular mutational heterogeneity in colon cancer cells. Cancers (Basel) 11: 892. 10.3390/cancers11070892 31247990 PMC6678395

[bib15] Dubois F, Jean-Jacques B, Roberge H, Bénard M, Galas L, Schapman D, Elie N, Goux D, Keller M, Maille E, (2018) A role for RASSF1A in tunneling nanotube formation between cells through GEFH1/Rab11 pathway control. Cell Commun Signal 16: 66. 10.1186/s12964-018-0276-4 30305100 PMC6180646

[bib16] D’Aloia A, Arrigoni E, Costa B, Berruti G, Martegani E, Sacco E, Ceriani M (2021) RalGPS2 interacts with Akt and PDK1 promoting tunneling nanotubes formation in bladder cancer and kidney cells microenvironment. Cancers (Basel) 13: 6330. 10.3390/cancers13246330 34944949 PMC8699646

[bib17] Franchi M, Piperigkou Z, Riti E, Masola V, Onisto M, Karamanos NK (2020) Long filopodia and tunneling nanotubes define new phenotypes of breast cancer cells in 3D cultures. Matrix Biol Plus 6-7: 100026. 10.1016/j.mbplus.2020.100026 33543024 PMC7852320

[bib18] Gherardi E, Sandin S, Petoukhov MV, Finch J, Youles ME, Öfverstedt L-G, Miguel RN, Blundell TL, Vande Woude GF, Skoglund U, (2006) Structural basis of hepatocyte growth factor/scatter factor and MET signalling. Proc Natl Acad Sci U S A 103: 4046–4051. 10.1073/pnas.0509040103 16537482 PMC1449643

[bib19] González MN, De Mello W, Butler-Browne GS, Silva-Barbosa SD, Mouly V, Savino W, Riederer I (2017) HGF potentiates extracellular matrix-driven migration of human myoblasts: Involvement of matrix metalloproteinases and MAPK/ERK pathway. Skelet Muscle 7: 20. 10.1186/s13395-017-0138-6 29017538 PMC5635537

[bib20] Gousset K, Marzo L, Commere P-H, Zurzolo C (2013) Myo10 is a key regulator of TNT formation in neuronal cells. J Cell Sci 126: 4424–4435. 10.1242/jcs.129239 23886947

[bib21] Hanna SJ, McCoy-Simandle K, Miskolci V, Guo P, Cammer M, Hodgson L, Cox D (2017) The role of Rho-GTPases and actin polymerization during macrophage tunneling nanotube biogenesis. Sci Rep 7: 8547–8616. 10.1038/s41598-017-08950-7 28819224 PMC5561213

[bib22] Hanna SJ, McCoy-Simandle K, Leung E, Genna A, Condeelis J, Cox D (2019) Tunneling nanotubes, a novel mode of tumor cell–macrophage communication in tumor cell invasion. J Cell Sci 132: jcs223321. 10.1242/jcs.223321 30659112 PMC6382011

[bib23] Hase K, Kimura S, Takatsu H, Ohmae M, Kawano S, Kitamura H, Ito M, Watarai H, Hazelett CC, Yeaman C, (2009) M-Sec promotes membrane nanotube formation by interacting with Ral and the exocyst complex. Nat Cell Biol 11: 1427–1432. 10.1038/ncb1990 19935652

[bib24] Ichimura E, Maeshima A, Nakajima T, Nakamura T (1996) Expression of c‐met/HGF receptor in human non‐small cell lung carcinomas in vitro and in vivo and its prognostic significance. Jpn J Cancer Res 87: 1063–1069. 10.1111/j.1349-7006.1996.tb03111.x 8957065 PMC5920996

[bib25] Ishibe S, Joly D, Zhu X, Cantley LG (2003) Phosphorylation-dependent paxillin-ERK association mediates hepatocyte growth factor-stimulated epithelial morphogenesis. Mol Cell 12: 1275–1285. 10.1016/s1097-2765(03)00406-4 14636584

[bib26] Ishibe S, Joly D, Liu Z-X, Cantley LG (2004) Paxillin serves as an ERK-regulated scaffold for coordinating FAK and Rac activation in epithelial morphogenesis. Mol Cell 16: 257–267. 10.1016/j.molcel.2004.10.006 15494312

[bib27] Jahangiri A, Nguyen A, Chandra A, Sidorov MK, Yagnik G, Rick J, Han SW, Chen W, Flanigan PM, Schneidman-Duhovny D, (2017) Cross-activating c-Met/β1 integrin complex drives metastasis and invasive resistance in cancer. Proc Natl Acad Sci U S A 114: E8685–E8694. 10.1073/pnas.1701821114 28973887 PMC5642678

[bib28] Jana A, Ladner K, Lou E, Nain AS (2022) Tunneling nanotubes between cells migrating in ECM mimicking fibrous environments. Cancers (Basel) 14: 1989. 10.3390/cancers14081989 35454893 PMC9030013

[bib29] Ju L, Zhou C (2013) Association of integrin beta1 and c-MET in mediating EGFR TKI gefitinib resistance in non-small cell lung cancer. Cancer Cell Int 13: 15. 10.1186/1475-2867-13-15 23402326 PMC3583715

[bib30] Kermorgant S, Aparicio T, Dessirier V, Lewin MJ, Lehy T (2001) Hepatocyte growth factor induces colonic cancer cell invasiveness via enhanced motility and protease overproduction. Evidence for PI3 kinase and PKC involvement. Carcinogenesis 22: 1035–1042. 10.1093/carcin/22.7.1035 11408346

[bib31] Kermorgant S, Zicha D, Parker PJ (2004) PKC controls HGF‐dependent c‐Met traffic, signalling and cell migration. EMBO J 23: 3721–3734. 10.1038/sj.emboj.7600396 15385963 PMC522795

[bib32] Kolba MD, Dudka W, Zaręba-Kozioł M, Kominek A, Ronchi P, Turos L, Chroscicki P, Wlodarczyk J, Schwab Y, Klejman A, (2019) Tunneling nanotube-mediated intercellular vesicle and protein transfer in the stroma-provided imatinib resistance in chronic myeloid leukemia cells. Cell Death Dis 10: 817. 10.1038/s41419-019-2045-8 31659149 PMC6817823

[bib33] Kretschmer A, Zhang F, Somasekharan SP, Tse C, Leachman L, Gleave A, Li B, Asmaro I, Huang T, Kotula L, (2019) Stress-induced tunneling nanotubes support treatment adaptation in prostate cancer. Sci Rep 9: 7826–7913. 10.1038/s41598-019-44346-5 31127190 PMC6534589

[bib34] Kumar A, Kim JH, Ranjan P, Metcalfe MG, Cao W, Mishina M, Gangappa S, Guo Z, Boyden ES, Zaki S, (2017) Influenza virus exploits tunneling nanotubes for cell-to-cell spread. Sci Rep 7: 40360–40414. 10.1038/srep40360 28059146 PMC5216422

[bib35] Lai J-F, Kao S-C, Jiang S-T, Tang M-J, Chan P-C, Chen H-C (2000) Involvement of focal adhesion kinase in hepatocyte growth factor-induced scatter of Madin-Darby canine kidney cells. J Biol Chem 275: 7474–7480. 10.1074/jbc.275.11.7474 10713050

[bib36] Lau D, Wadhwa H, Sudhir S, Chang AC-C, Jain S, Chandra A, Nguyen AT, Spatz JM, Pappu A, Shah SS, (2021) Role of c-Met/β1 integrin complex in the metastatic cascade in breast cancer. JCI Insight 6: e138928. 10.1172/jci.insight.138928 34003803 PMC8262466

[bib37] Laukaitis CM, Webb DJ, Donais K, Horwitz AF (2001) Differential dynamics of alpha 5 integrin, paxillin, and alpha-actinin during formation and disassembly of adhesions in migrating cells. J Cell Biol 153: 1427–1440. 10.1083/jcb.153.7.1427 11425873 PMC2150721

[bib38] Lin T-K, Chen S-D, Chuang Y-C, Lan M-Y, Chuang J-H, Wang P-W, Hsu T-Y, Wang F-S, Tsai M-H, Huang S-T (2019) Mitochondrial transfer of Wharton's jelly mesenchymal stem cells eliminates mutation burden and rescues mitochondrial bioenergetics in rotenone-stressed MELAS fibroblasts. Oxid Med Cell Longev 2019: 9537504. 10.1155/2019/9537504 31249652 PMC6556302

[bib39] Liu Z-X, Yu CF, Nickel C, Thomas S, Cantley LG (2002) Hepatocyte growth factor induces ERK-dependent paxillin phosphorylation and regulates paxillin-focal adhesion kinase association. J Biol Chem 277: 10452–10458. 10.1074/jbc.M107551200 11784715

[bib40] Lou E, Fujisawa S, Morozov A, Barlas A, Romin Y, Dogan Y, Gholami S, Moreira AL, Manova-Todorova K, Moore MA (2012) Tunneling nanotubes provide a unique conduit for intercellular transfer of cellular contents in human malignant pleural mesothelioma. PLoS One 7: e33093. 10.1371/journal.pone.0033093 22427958 PMC3302868

[bib41] Lu J, Zheng X, Li F, Yu Y, Chen Z, Liu Z, Wang Z, Xu H, Yang W (2017) Tunneling nanotubes promote intercellular mitochondria transfer followed by increased invasiveness in bladder cancer cells. Oncotarget 8: 15539–15552. 10.18632/oncotarget.14695 28107184 PMC5362504

[bib42] Lu JJ, Yang WM, Li F, Zhu W, Chen Z (2019) Tunneling nanotubes mediated microRNA-155 intercellular transportation promotes bladder cancer cells' invasive and proliferative capacity. Int J Nanomedicine 14: 9731–9743. 10.2147/IJN.S217277 31849465 PMC6911338

[bib43] Ma PC, Maulik G, Christensen J, Salgia R (2003) c-Met: structure, functions and potential for therapeutic inhibition. Cancer Metastasis Rev 22: 309–325. 10.1023/a:1023768811842 12884908

[bib44] Masuya D, Huang C, Liu D, Nakashima T, Kameyama K, Haba R, Ueno M, Yokomise H (2004) The tumour–stromal interaction between intratumoral c-Met and stromal hepatocyte growth factor associated with tumour growth and prognosis in non-small-cell lung cancer patients. Br J Cancer 90: 1555–1562. 10.1038/sj.bjc.6601718 15083185 PMC2409699

[bib45] McBain VA, Forrester JV, McCaig CD (2003) HGF, MAPK, and a small physiological electric field interact during corneal epithelial cell migration. Invest Ophthalmol Vis Sci 44: 540–547. 10.1167/iovs.02-0570 12556381

[bib46] Ménard L, Parker PJ, Kermorgant S (2014) Receptor tyrosine kinase c-Met controls the cytoskeleton from different endosomes via different pathways. Nat Commun 5: 3907. 10.1038/ncomms4907 24835487

[bib47] Mendoza MC, Er EE, Zhang W, Ballif BA, Elliott HL, Danuser G, Blenis J (2011) ERK-MAPK drives lamellipodia protrusion by activating the WAVE2 regulatory complex. Mol Cell 41: 661–671. 10.1016/j.molcel.2011.02.031 21419341 PMC3078620

[bib48] Mitra A, Sawada K, Tiwari P, Mui K, Gwin K, Lengyel E (2011) Ligand-independent activation of c-Met by fibronectin and α5β1-integrin regulates ovarian cancer invasion and metastasis. Oncogene 30: 1566–1576. 10.1038/onc.2010.532 21119598 PMC3069218

[bib49] Molina JR, Yang P, Cassivi SD, Schild SE, Adjei AA (2008) Non-small cell lung cancer: Epidemiology, risk factors, treatment, and survivorship. In Mayo clinic proceedings, pp 584–594 Amsterdam: Elsevier. 10.4065/83.5.584 PMC2718421 18452692

[bib50] Nakamura Y, Niki T, Goto A, Morikawa T, Miyazawa K, Nakajima J, Fukayama M (2007) c‐Met activation in lung adenocarcinoma tissues: an immunohistochemical analysis. Cancer Sci 98: 1006–1013. 10.1111/j.1349-7006.2007.00493.x 17459054 PMC11159971

[bib51] Ohmichi H, Matsumoto K, Nakamura T (1996) In vivo mitogenic action of HGF on lung epithelial cells: Pulmotrophic role in lung regeneration. Am J Physiol 270: L1031–L1039. 10.1152/ajplung.1996.270.6.L1031 8764230

[bib52] Olivero M, Rizzo M, Madeddu R, Casadio C, Pennacchietti S, Nicotra M, Prat M, Maggi G, Arena N, Natali PG, (1996) Overexpression and activation of hepatocyte growth factor/scatter factor in human non-small-cell lung carcinomas. Br J Cancer 74: 1862–1868. 10.1038/bjc.1996.646 8980383 PMC2074802

[bib53] Osteikoetxea-Molnár A, Szabó-Meleg E, Tóth EA, Oszvald Á, Izsépi E, Kremlitzka M, Biri B, Nyitray L, Bozó T, Németh P, (2016) The growth determinants and transport properties of tunneling nanotube networks between B lymphocytes. Cell Mol Life Sci 73: 4531–4545. 10.1007/s00018-016-2233-y 27125884 PMC11108537

[bib54] Onfelt B, Nedvetzki S, Benninger RK, Purbhoo MA, Sowinski S, Hume AN, Seabra MC, Neil MA, French PM, Davis DM (2006) Structurally distinct membrane nanotubes between human macrophages support long-distance vesicular traffic or surfing of bacteria. J Immunol 177: 8476–8483. 10.4049/jimmunol.177.12.8476 17142745

[bib55] Pasquier J, Guerrouahen BS, Al Thawadi H, Ghiabi P, Maleki M, Abu-Kaoud N, Jacob A, Mirshahi M, Galas L, Rafii S, (2013) Preferential transfer of mitochondria from endothelial to cancer cells through tunneling nanotubes modulates chemoresistance. J Transl Med 11: 94. 10.1186/1479-5876-11-94 23574623 PMC3668949

[bib56] Porter L, Minaisah R-M, Ahmed S, Ali S, Norton R, Zhang Q, Ferraro E, Molenaar C, Holt M, Cox S, (2020) SUN1/2 are essential for RhoA/ROCK-regulated actomyosin activity in isolated vascular smooth muscle cells. Cells 9: 132. 10.3390/cells9010132 31935926 PMC7017107

[bib57] Puri N, Salgia R (2008) Synergism of EGFR and c-Met pathways, cross-talk and inhibition, in non-small cell lung cancer. J Carcinog 7: 9. 10.4103/1477-3163.44372 19240370 PMC2669728

[bib58] Roehlecke C, Schmidt MH (2020) Tunneling nanotubes and tumor microtubes in cancer. Cancers 12: 857. 10.3390/cancers12040857 32244839 PMC7226329

[bib59] Rustom A, Saffrich R, Markovic I, Walther P, Gerdes H-H (2004) Nanotubular highways for intercellular organelle transport. Science 303: 1007–1010. 10.1126/science.1093133 14963329

[bib60] Sáenz-de-Santa-María I, Bernardo-Castiñeira C, Enciso E, García-Moreno I, Chiara JL, Suarez C, Chiara M-D (2017) Control of long-distance cell-to-cell communication and autophagosome transfer in squamous cell carcinoma via tunneling nanotubes. Oncotarget 8: 20939–20960. 10.18632/oncotarget.15467 28423494 PMC5400557

[bib61] Sastry SK, Lakonishok M, Wu S, Truong TQ, Huttenlocher A, Turner CE, Horwitz AF (1999) Quantitative changes in integrin and focal adhesion signaling regulate myoblast cell cycle withdrawal. J Cell Biol 144: 1295–1309. 10.1083/jcb.144.6.1295 10087271 PMC2150582

[bib62] Schaller MD, Otey CA, Hildebrand JD, Parsons JT (1995) Focal adhesion kinase and paxillin bind to peptides mimicking beta integrin cytoplasmic domains. J Cell Biol 130: 1181–1187. 10.1083/jcb.130.5.1181 7657702 PMC2120552

[bib63] Scheiblich H, Dansokho C, Mercan D, Schmidt SV, Bousset L, Wischhof L, Eikens F, Odainic A, Spitzer J, Griep A, (2021) Microglia jointly degrade fibrillar alpha-synuclein cargo by distribution through tunneling nanotubes. Cell 184: 5089–5106.e21. 10.1016/j.cell.2021.09.007 34555357 PMC8527836

[bib64] Schiller C, Diakopoulos KN, Rohwedder I, Kremmer E, von Toerne C, Ueffing M, Weidle UH, Ohno H, Weiss EH (2013) LST1 promotes the assembly of a molecular machinery responsible for tunneling nanotube formation. J Cell Sci 126: 767–777. 10.1242/jcs.114033 23239025

[bib65] Singh-Kaw P, Zarnegar R, Siegfried JM (1995) Stimulatory effects of hepatocyte growth factor on normal and neoplastic human bronchial epithelial cells. Am J Physiol 268: L1012–L1020. 10.1152/ajplung.1995.268.6.L1012 7611423

[bib66] Sonnenberg E, Meyer D, Weidner KM, Birchmeier C (1993) Scatter factor/hepatocyte growth factor and its receptor, the c-met tyrosine kinase, can mediate a signal exchange between mesenchyme and epithelia during mouse development. J Cell Biol 123: 223–235. 10.1083/jcb.123.1.223 8408200 PMC2119804

[bib67] Spees JL, Olson SD, Whitney MJ, Prockop DJ (2006) Mitochondrial transfer between cells can rescue aerobic respiration. Proc Natl Acad Sci U S A 103: 1283–1288. 10.1073/pnas.0510511103 16432190 PMC1345715

[bib68] Stamenkovic I (2003) Extracellular matrix remodelling: The role of matrix metalloproteinases. J Pathol 200: 448–464. 10.1002/path.1400 12845612

[bib69] Stoker M, Gherardi E, Perryman M, Gray J (1987) Scatter factor is a fibroblast-derived modulator of epithelial cell mobility. Nature 327: 239–242. 10.1038/327239a0 2952888

[bib70] Syed ZA, Yin W, Hughes K, Gill JN, Shi R, Clifford JL (2011) HGF/c-met/Stat3 signaling during skin tumor cell invasion: Indications for a positive feedback loop. BMC Cancer 11: 180–211. 10.1186/1471-2407-11-180 21595927 PMC3112164

[bib71] Thayanithy V, Dickson EL, Steer C, Subramanian S, Lou E (2014) Tumor-stromal cross talk: Direct cell-to-cell transfer of oncogenic microRNAs via tunneling nanotubes. Transl Res 164: 359–365. 10.1016/j.trsl.2014.05.011 24929208 PMC4242806

[bib72] To Y, Dohi M, Matsumoto K, Tanaka R, Sato A, Nakagome K, Nakamura T, Yamamoto K (2002) A two-way interaction between hepatocyte growth factor and interleukin-6 in tissue invasion of lung cancer cell line. Am J Respir Cell Mol Biol 27: 220–226. 10.1165/ajrcmb.27.2.4804 12151314

[bib73] Tretiakova M, Salama AK, Karrison T, Ferguson MK, Husain AN, Vokes EE, Salgia R (2011) MET and phosphorylated MET as potential biomarkers in lung cancer. J Environ Pathol Toxicol Oncol 30: 341–354. 10.1615/jenvironpatholtoxicoloncol.v30.i4.70 22181983

[bib74] Wang X, Gerdes H-H (2015) Transfer of mitochondria via tunneling nanotubes rescues apoptotic PC12 cells. Cell Death Differ 22: 1181–1191. 10.1038/cdd.2014.211 25571977 PMC4572865

[bib75] Wang Y, Cui J, Sun X, Zhang Y (2011) Tunneling-nanotube development in astrocytes depends on p53 activation. Cell Death Differ 18: 732–742. 10.1038/cdd.2010.147 21113142 PMC3131904

[bib76] Wang Z-G, Liu S-L, Tian Z-Q, Zhang Z-L, Tang H-W, Pang D-W (2012) Myosin-driven intercellular transportation of wheat germ agglutinin mediated by membrane nanotubes between human lung cancer cells. ACS Nano 6: 10033–10041. 10.1021/nn303729r 23102457

[bib77] Wang J, Liu X, Qiu Y, Shi Y, Cai J, Wang B, Wei X, Ke Q, Sui X, Wang Y, (2018) Cell adhesion-mediated mitochondria transfer contributes to mesenchymal stem cell-induced chemoresistance on T cell acute lymphoblastic leukemia cells. J Hematol Oncol 11: 11–13. 10.1186/s13045-018-0554-z 29357914 PMC5778754

[bib78] Wang F, Chen X, Cheng H, Song L, Liu J, Caplan S, Zhu L, Wu JY (2021) MICAL2PV suppresses the formation of tunneling nanotubes and modulates mitochondrial trafficking. EMBO Rep 22: e52006. 10.15252/embr.202052006 34096155 PMC8366454

[bib79] Yanagita K, Matsumoto K, Sekiguchi K, Ishibashi H, Niho Y, Nakamura T (1993) Hepatocyte growth factor may act as a pulmotrophic factor on lung regeneration after acute lung injury. J Biol Chem 268: 21212–21217. 10.1016/s0021-9258(19)36912-1 8407957

[bib80] Zhang YW, Vande Woude GF (2003) HGF/SF‐met signaling in the control of branching morphogenesis and invasion. J Cell Biochem 88: 408–417. 10.1002/jcb.10358 12520544

[bib81] Zhu D, Tan KS, Zhang X, Sun AY, Sun GY, Lee JC-M (2005) Hydrogen peroxide alters membrane and cytoskeleton properties and increases intercellular connections in astrocytes. J Cell Sci 118: 3695–3703. 10.1242/jcs.02507 16046474

